# GDF15 Neutralization Ameliorates Muscle Atrophy and Exercise Intolerance in a Mouse Model of Mitochondrial Myopathy

**DOI:** 10.1002/jcsm.13715

**Published:** 2025-02-20

**Authors:** Stephen E. Flaherty, LouJin Song, Bina Albuquerque, Anthony Rinaldi, Mary Piper, Dinesh Hirenallur Shanthappa, Xian Chen, John Stansfield, Shoh Asano, Evanthia Pashos, Trenton Thomas Ross, Srinath Jagarlapudi, Abdul Sheikh, Bei Zhang, Zhidan Wu

**Affiliations:** ^1^ Internal Medicine Research Unit Pfizer Worldwide Research, Development & Medical Cambridge Massachusetts USA; ^2^ Obesity and Complications Eli Lilly Boston Massachusetts USA; ^3^ Diabetes, Obesity and MASH, Global Drug Discovery Novo Nordisk Lexington Massachusetts USA; ^4^ Program Mamager, Preclinical Sciences, Toxicology Vertex Pharmaceuticals Boston Massachusetts USA; ^5^ Biostatistics, Early Clinical Development Pfizer Worldwide Research, Development & Medical Cambridge Massachusetts USA; ^6^ Inflammation and Immunology Research Unit Pfizer Worldwide Research, Development & Medical Cambridge Massachusetts USA

**Keywords:** antibody, GDF15, mice, mitochondria, muscle, primary mitochondrial myopathy

## Abstract

**Background:**

Primary mitochondrial myopathies (PMMs) are disorders caused by mutations in genes encoding mitochondrial proteins and proteins involved in mitochondrial function. PMMs are characterized by loss of muscle mass and strength as well as impaired exercise capacity. Growth/Differentiation Factor 15 (GDF15) was reported to be highly elevated in PMMs and cancer cachexia. Previous studies have shown that GDF15 neutralization is effective in improving skeletal muscle mass and function in cancer cachexia. It remains to be determined if the inhibition of GDF15 could be beneficial for PMMs. The purpose of the present study is to assess whether treatment with a GDF15 neutralizing antibody can alleviate muscle atrophy and physical performance impairment in a mouse model of PMM.

**Methods:**

The effects of GDF15 neutralization on PMM were assessed using Polg^D257A/D257A^ (POLG) mice. These mice express a proofreading‐deficient version of the mitochondrial DNA polymerase gamma, leading to an increased rate of mutations in mitochondrial DNA (mtDNA). These animals display increased circulating GDF15 levels, reduced muscle mass and function, exercise intolerance, and premature aging. Starting at 9 months of age, the mice were treated with an anti‐GDF15 antibody (mAB2) once per week for 12 weeks. Body weight, food intake, body composition, and muscle mass were assessed. Muscle function and exercise capacity were evaluated using in vivo concentric max force stimulation assays, forced treadmill running and voluntary home‐cage wheel running. Mechanistic investigations were performed via muscle histology, bulk transcriptomic analysis, RT‐qPCR and western blotting.

**Results:**

Anti‐GDF15 antibody treatment ameliorated the metabolic phenotypes of the POLG animals, improving body weight (+13% ± 8%, *p* < 0.0001), lean mass (+13% ± 15%, *p* < 0.001) and muscle mass (+35% ± 24%, *p* < 0.001). Additionally, the treatment improved skeletal muscle max force production (+35% ± 43%, *p* < 0.001) and exercise performance, including treadmill (+40% ± 29%, *p* < 0.05) and voluntary wheel running (+320% ± 19%, *p* < 0.05). Mechanistically, the beneficial effects of GDF15 neutralization are linked to the reversal of the transcriptional dysregulation in genes involved in autophagy and proteasome signalling. The treatment also appears to dampen glucocorticoid signalling by suppressing circulating corticosterone levels in the POLG animals.

**Conclusions:**

Our findings highlight the potential of GDF15 neutralization with a monoclonal antibody as a therapeutic avenue to enhance physical performance and mitigate adverse clinical outcomes in patients with PMM.

## 1 Introduction

Primary mitochondrial myopathies (PMMs) are genetic disorders caused by pathogenic mutations in genes in mitochondrial DNA (mtDNA) and nuclear DNA (nDNA) that encode mitochondrial proteins or proteins involved in mitochondrial function [1]. PMMs affect predominantly, but not exclusively, skeletal muscle [2]. The most common symptoms are muscle weakness, exercise intolerance and progressive external ophthalmoplegia [3]. The prevalence of PMM is estimated to be 1:4300 individuals, with most displaying prominent muscle abnormalities [3] (Supporting Information: Reference S1). There are currently no approved therapies for PMM and new therapies that may alleviate muscle abnormalities and improve physical performance in PMM are needed.

Mitochondria play a central role in ATP production, fatty acid oxidation, ROS generation and apoptosis activation [4] (Supporting Information: Reference S2). The mitochondrion contains its own DNA, encoding many key players in mitochondrial function and health, that operates independently of nuclear regulation and repair processes. Mutations in mtDNA have been shown to accumulate in a variety of tissues in humans, nonhuman primates and rodents as they age [5, 6] (Supporting Information: References S3–S6). An overwhelming abundance of mtDNA mutations causes mitochondrial dysfunction, resulting in phenotypes associated with aging, such as muscle loss and weakness [7] (Supporting Information: Reference S7).

Several pathways and factors have been implicated in contributing to muscle atrophy, weakness and impairment of physical performance resulting from pathogenic mutations encoded in mitochondrial proteins in preclinical models of mitochondrial myopathy. These perturbed pathways include, but are not limited to, the ubiquitin proteasome system (UPS) and autophagy/mitophagy, defects in oxidative phosphorylation, imbalance in mitochondrial dynamics and biosynthesis, inflammation and immune response, cellular stress and metabolic disturbances [8]. Forkhead box O3 (FoxO3), peroxisome proliferator‐activated receptors (PPARs), adenosine monophosphate‐activated protein kinase (AMPK), sirtuins and fibroblast growth factor 21 (FGF21) are known regulators of some of these perturbed pathways [8] (Supporting Information: Reference S8). Interestingly, elevated circulating FGF21 and growth differentiation factor 15 (GDF15) levels were reported in PMM patients and preclinical mouse models of mitochondrial myopathy [1, 9] (Supporting Information: References S9 and S10). Deletion of FGF21 appeared to be beneficial in alleviating the disease condition in OPA1 KO mice [10], a model of mitochondrial myopathy.

GDF15 is an inflammatory cytokine released in response to cellular stress [11], and it is implicated as a key driver of anorexia and weight loss in nonhuman primates and rodents [12] (Supporting Information: References S11–S16). Several lines of evidence support GDF15 as a key driver of anorexia, muscle wasting and weight loss, including the following: (1) Increased circulating levels of GDF15 are associated with weight loss in cancer patients and poor survival in many chronic diseases [13] (Supporting Information: References S17–S23), (2) GDF15 neutralization reversed anorexia, weight loss and skeletal muscle function impairment in preclinical cachexia models [14, 15, 16] (Supporting Information: References S24 and S25), and (3) ponsegromab, an anti‐GDF15 antibody, demonstrated significant benefits in cancer cachexia patients in a phase 2 clinical study (NCT05546476), inducing weight gain, improving physical activity and reducing cachexia symptoms in these patients (Supporting Information: Reference S26).

Given the significant role of GDF15 in driving muscle wasting and physical activities in cancer cachexia, coupled with the marked increase of circulating levels of GDF15 in PMM patients, it is plausible GDF15 plays a role in mediating the pathways implicated in PMM. Additionally, GDF15 neutralization could be a potential therapeutic avenue for patients with PMM. The purpose of the present study is to assess whether treatment with a GDF15 neutralizing antibody can alleviate muscle atrophy and physical performance impairment in POLG mutator mice, a mouse model of PMM.

## 2 Materials and Methods

### 2.1 Animals

Male Wild Type (WT) and Homozygous (Polg^D257A/D257A^) mtDNA mutator (POLG) mice were obtained from Jackson Laboratory (Stock No. 017341). All mice were individually housed at thermoneutral conditions (27 ± 1°C) and maintained on a standard light–dark cycle (6 AM to 6 PM). They were allowed ad libitum access to water and food (Purina rodent diet 5061; Purina Mills, St. Louis, MO, USA) except when specified for food intake measurements. All procedures were approved by the Pfizer Groton and Cambridge Animal Care and Use Committees in accordance with the ethical standard laid down in the 1964 Declaration of Helsinki and its later amendments.

Body weight was measured weekly (Mettler Toledo ME4002TE, Mettler Toledo, Oakland, CA). Body composition was assessed using the EchoMRI 4‐in‐1500 Body Composition Analyzer (EchoMRI, Houston, TX).

### 2.2 Plasma GDF15, FGF21 and Corticosterone Measurement

Tail blood samples were collected from 3‐, 6‐ and 10‐month‐old WT and POLG mice and from all three groups in the intervention study at day 87 post‐initiation of treatment. Plasma GDF‐15, FGF21 and corticosterone levels were measured using ELISA kits from R&D Systems (Cat# MGD150 for GDF‐15, MF2100 for FGF‐21 and KGE009 for corticosterone R&D Systems, Minneapolis, MN, USA). The assays were performed following the manufacturer's instructions and protocols.

### 2.3 Voluntary Wheel Running Measurement

To measure the voluntary wheel running activity, mice were housed in thermoneutral conditions with free access to the wheels (Columbus Instruments, Chicago, IL, USA), food and water ad libitum on a standard light–dark cycle (6 AM–6 PM). The wheel counts, indicative of running distance, were measured daily for 5 days after an acclimation period of 3 days with access to voluntary wheel running.

### 2.4 In Vivo Muscle Force Generation Measurement

Mice were anaesthetized with 2% isoflurane and placed supine on a platform heated via a circulating water bath at 37°C. The right leg was shaved up to the patella and right knee stabilized via knee clamp. Once stabilized, the right foot was affixed to a Dual Mode Foot Plate (300‐C FP, Aurora Scientific Inc., Aurora, Canada), and two electrodes were placed subcutaneously near the mid‐belly of the gastrocnemius to achieve plantar flexion. A 1‐Hz electrical stimulation was delivered (0.2‐s duration, 1 s between stimulations) via stimulator (701C, Aurora Scientific Inc.) while increasing amperes to 50, 100 and 150 Hz to generate a maximum twitch measurement. All data were collected and analysed using the manufacturer supplied software (DMC and DMA, Aurora Scientific Inc.). Mice were housed at thermoneutral temperature prior to analysis.

### 2.5 Treadmill Running Assessment

All mice were acclimated for 2 days before the testing day. The first acclimation day consisted of placing the mice on the nonmoving belt for 25 min before allowing mice to run for 5 min at 3 m/min. The following day all mice were acclimated to the non‐moving belt for 20 min before allowing the mice to run for 5 min at 3 m/min. On testing day, mice were placed on the non‐moving treadmill for 20 min before allowing the mice to run for 2 min at 8 m/min. Speed was increased to 12 m/min for two more minutes followed by an increase of 3 m/min every 2 min until exhaustion where mice remained on shock grid for three consecutive seconds for three subsequent instances. All testing was performed on the Modular Enclosed Metabolic Treadmill for Mice (four lanes total) from Columbus Instruments with the shock grid set to 2 Hz with a 10° incline (http://www.colinst.com/products/metabolic‐modular‐treadmill).

### 2.6 Anti‐GDF15 mAB2 Intervention

The anti‐GDF15 antibody mAB2 is a GDF15 specific antibody generated at Pfizer with high‐affinity binding to both mouse and human GDF15. The characterization of the antibody was reported previously [14, 15, 16]. POLG mice (9‐month‐old) were randomized into two groups: POLG‐Veh and POLG‐GDF15 mAB2. WT littermates were also included in the study and dosed with control IgG antibody. Mice were dosed subcutaneously with either 10 mg/kg of anti‐GDF15 mAB2 antibody (POLG‐GDF15 mAB2) or control IgG antibody (WT‐Veh and POLG‐Veh) once per week through the duration of the study (12 weeks). Body weight was measured weekly. Fat and lean mass were assessed using EchoMRI 4100 system.

### 2.7 Histology

Mouse gastrocnemius and soleus muscle complex was isolated, mounted on a cork and snap‐frozen in cold isopentane (Sigma‐Aldrich, 277 258) for cryo‐sectioning. Cross‐sections of 10‐μm thickness were collected from the belly of the samples and placed on Superfrost Plus microscope slides. For fibre size evaluation, the sections were fixed with 10% neutral‐buffered formalin (NBF) for 10 mins at room temperature, stained with WGA647 (ThermoFisher, W32466) for 1 h under room temperature, mounted with VECTASHIELD Antifade Mounting Medium with 4′,6‐diamidino‐2‐phenylindole (DAPI) (Vector Laboratories, H‐1800) and were imaged using a Zeiss AxioScan Z.1 slide scanner (Carl Zeiss, Jena, Germany). The images were analysed using Visiopharm (Version 2020.09.0.8195) and custom‐designed applications. The fibre size was the median cross‐sectional area of all fibres in the cross‐section.

### 2.8 RNAseq Sample Preparation

Gastrocnemius muscle was collected from WT‐Veh, POLG‐Veh and POLG‐GDF15 mAB2 animals at 12 months of age, after 12 weeks of antibody treatment and snap‐frozen in liquid nitrogen. Tissue was pulverized and total RNA was extracted using Qiagen Rneasy Plus Universal mini kit following manufacturer's instructions (Qiagen, Hilden, Germany). The RNA samples were quantified using the Qubit 2.0 Fluorometer (ThermoFisher Scientific, Waltham, MA, USA) and RNA integrity was evaluated using the TapeStation electrophoresis system (Agilent Technologies, Palo Alto, CA, USA). RNA sequencing libraries were prepared using the NEBNext Ultra Directional RNA Library Prep Kit from Illumina following manufacturer's instructions (NEB, Ipswich, MA, USA). Briefly, mRNAs were first enriched with Oligo (dT) beads. Enriched mRNAs were fragmented for 15 min at 94°C. First‐strand and second‐strand cDNAs were subsequently synthesized. cDNA fragments were end repaired and adenylated at 3′ ends, and universal adapters were ligated to cDNA fragments, followed by index addition and library enrichment by limited‐cycle PCR. The sequencing libraries were validated on the Agilent TapeStation (Agilent Technologies, Palo Alto, CA, USA) and quantified by using Qubit 2.0 Fluorometer (Invitrogen, Carlsbad, CA) as well as by quantitative PCR (KAPA Biosystems, Wilmington, MA, USA).

### 2.9 RNA Sequencing

The sequencing libraries were multiplexed and clustered onto an Illumina flowcell. After clustering, the flow cell was loaded onto the Illumina NovaSeq 6000 instrument according to manufacturer's instructions. The samples were sequenced using a 2x150bp Paired End (PE) configuration and targeting 30 million reads/sample. Image analysis and base calling were conducted by the Illumina Control Software (HCS). Raw sequence data (.bcl files) generated from Illumina were converted into fastq files and de‐multiplexed using Illumina bcl2fastq 2.20 software. One mis‐match was allowed for index sequence identification.

### 2.10 Transcriptomic Analysis

The raw FASTQ files were processed using an internal pipeline, Rnaseq Experiment DAshboard, utilizing STAR (v2.7.3a) (Supporting Information: Reference S27) and Salmon (v1.5.2) (Supporting Information: Reference S28) to map reads to the mouse genome and transcriptome (GRCm39, Ensembl release 106), respectively, with ERCC spike‐in sequences included. Quality of the samples was assessed using FastQC (v0.11.9), Picard Tools CollectRnaSeqMetrics (v 2.26.0) [17] and MultiQC (v1.11) (Supporting Information: Reference S29) to explore base quality, rRNA content, intronic percentages and mapping rates. The Salmon transcript abundance estimates were aggregated to the gene level using tximport (v1.22.0) (Supporting Information: Reference S30). Additional quality control was performed on the gene‐level counts using principal component analysis. Differential expression analysis was performed with DESeq2 (v1.34.0) (Supporting Information: Reference S31), whereas gene set enrichment analysis (GSEA) of the differential expression results was executed using clusterProfiler (v4.2.2) (Supporting Information: Reference S32) with KEGG (Supporting Information: References S33–S35) and MSigDB (Supporting Information: References S36 and S37) gene sets. GSEA was performed on the test statistics output from DESeq2.

### 2.11 Quantitative RT‐PCR

Tibialis anterior muscle was collected from WT‐Veh, POLG‐Veh and POLG‐GDF15 mAB2 animals at 12 months of age, after 12 weeks of antibody treatment and snap‐frozen in liquid nitrogen. Ribonucleic acid (RNA) was extracted and purified using RNeasy RNA Isolation Kit (QIAGEN, Germantown, MD), and then reverse transcribed into complementary deoxyribonucleic acid (cDNA) with a High‐Capacity cDNA Reverse Transcription Kit (Applied Biosystems, Foster City, CA). Quantitative reverse transcriptase polymerase chain reaction (qRT‐PCR) was performed using TaqMan reagents and primer‐probes (Applied Biosystems, Foster City, CA). Gene expression was normalized to the control gene TATA box binding protein (Tbp). Probes were purchased from Thermo Fisher Scientific (Waltham, MA) with the following catalogue numbers: Trim63 (Mm01185221_m1), Fbxo32 (Mm00499523_m1), foxo3 (Mm01185722_m1), Gabarapl1 (Mm00457880_m1), Bnip3 (Mm01275600_g1), Map 1lc3b (Mm00782868_sH), Tbp (Mm01277042_m1).

### 2.12 Western Blotting

Tibialis anterior muscle was collected from WT‐Veh, POLG‐Veh and POLG‐GDF15 mAB2 animals at 12 months of age, after 12 weeks of antibody treatment and snap‐frozen in liquid nitrogen. Frozen tissues were homogenized in ice‐cold RIPA buffer (Sigma Aldrich, St. Louis, MO), and protein concentrations were determined using a BCA protein assay (Thermo Fisher Scientific, Waltham, MA). Protein extracts were separated on NuPAGE 4–20% Bis‐Tris gels (Bio‐Rad, Hercules, CA) and blotted onto PVDF membranes (Bio‐Rad, Hercules, CA). Membranes were blocked for 1 h at room temperature in TBST (0.1% Tween) containing 5% milk. Membranes were then incubated overnight at 4°C with primary antibodies. Following three washing steps with TBST (0.1% Tween), membranes were incubated with HRP‐conjugated secondary antibodies for 1 h at room temperature. After thorough washing, proteins were visualized with SuperSignal West Dura Extended Duration Substrate (Thermo Fisher Scientific, Waltham, MA) on the Bio‐Rad ChemiDoc MP. Immunoreactive bands were quantified using Image J Software (NIH). Primary antibodies used are Anti‐LC3B (Cell Signaling, #43566) and Anti‐Vinculin (ThermoFisher, #700062).

### 2.13 Echocardiography

Echocardiography was performed to assess left ventricular (LV) function and structure. The imaging was performed similar to previously published procedure in mouse (Supporting Information: Reference S47). Briefly, mice were anaesthetised using isoflurane (3%) in an induction chamber, and then anaesthetic status was maintained at ~2% isoflurane during animal preparation and image acquisition. Mice were prepared for imaging by removing hair around the left lateral and ventral thoracic area using a chemical hair removal agent. Then, the animal was transferred onto a heated (~34°C to 35°C) platform for echocardiography. Transthoracic parasternal long axis B‐mode, parasternal short axis M‐mode images of the left ventricle (LV) were acquired using 21‐MHz transducer (VisualSonics MX250S/MS‐250S®) in Vevo 2100® or 3100 ultrasound machine. Anaesthesia level was adjusted to maintain heart rate ~450 to 550 bpm during image acquisition. After image acquisition, the animal was transferred to a warm cage for recovery. Post image acquisition, images were analysed using Vevo lab® analysis software, and the following parameters were collected from image analysis: LV ejection fraction, LV dimensions and LV mass.

### 2.14 Statistical Analysis

The statistical analysis was conducted by a biostatistician. Data are expressed as mean ± standard deviation (SD). Statistical analyses and tests for significance for each measurement are described in the figure legends. Statistical significance is indicated in figures using the following denotation: *p < 0.05, **p < 0.01, ***p < 0.001, ****p < 0.0001.

## 3 Results

### 3.1 POLG Mutator Mice Demonstrated Elevated Circulating GDF15 Levels, Muscle Atrophy and Exercise Intolerance

mtDNA health is monitored by mitochondrial polymerase gamma (POLG) and POLG mutations are the most common cause of inherited mitochondrial disorders, with up to 2% of the population carrying these mutations [18]. Mice expressing a proofreading‐deficient version of the mitochondrial DNA polymerase gamma (POLG mutator mice) accumulate mtDNA mutations and exhibit features of PMM [19] (Supporting Information: Reference S11). Although young POLG animals are indistinguishable from WT littermates, long‐term observation reveals premature aging phenotypes including hair loss, greying and kyphosis. These animals have a reduced lifespan (< 50% of WT animals, ~48 weeks) and start demonstrating a weight loss phenotype around 24 weeks of age (6 months) [19]. Muscle atrophy and exercise impairment become apparent as these POLG animals age. However, longitudinal characterization of GDF15 circulating levels was not previously conducted in this model. Therefore, we first carried out a longitudinal characterization of GDF15 circulating levels and relevant phenotypes in the POLG mice to evaluate whether they exhibit elevated GDF15 levels and pathologies in skeletal muscle similar to patients with PMM.

**FIGURE 1:**
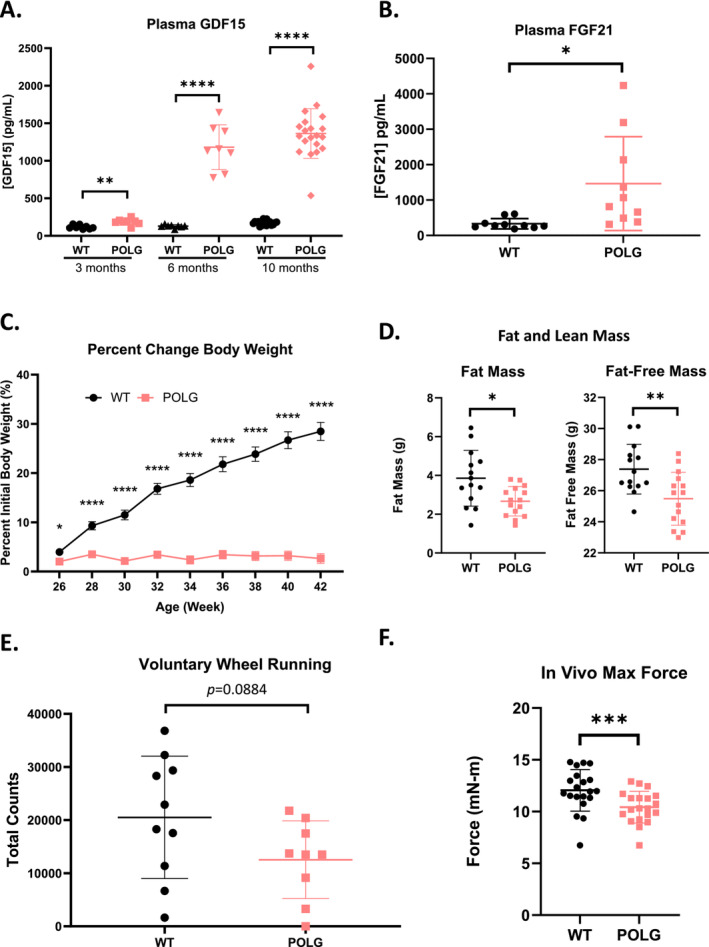
POLG mutator mice have elevated circulating GDF15 and FGF21 levels, body weight loss and exercise intolerance. (A) Plasma GDF15 levels in WT and POLG animals at 3 months, 6 months, and 10 months of age. *n* = 8 for WT and POLG at 3 and 6 months, *n* = 18 for WT and *n* = 20 for POLG at 10 months. Significance was assessed by Welch's two sample *t*‐test. (B) Plasma FGF21 levels in WT and POLG animals at 12 months of age. *n* = 10. Significance was assessed by Welch's two sample *t*‐test. The data were generated using plasma samples from WT‐Veh and POLG‐Veh groups and also shown in Figure 6F. (C) Percent change in body weight starting at 26 weeks of age. *n* = 20. Significance was assessed by one‐way ANOVA and Tukey's HSD. (D) Fat mass and fat‐free (lean) mass of WT and POLG animals at 6 months of age. *n* = 14 for WT and *n* = 15 for POLG. Significance was assessed by Welch's two sample *t*‐test. (E) Total wheel turns when given access to running wheels in the home cage. Assessment was conducted at 10 months of age. *n* = 10 for WT and *n* = 9 for POLG. Significance was assessed by Welch's two sample *t*‐test. (F) In vivo gastrocnemius/soleus complex muscle max concentric force generation in WT and POLG animals. Assessment was conducted at 10 months of age. *n* = 20/group. Significance was assessed by Welch's two sample *t*‐test. *p < 0.05, **p < 0.01, ***p < 0.001, ****p < 0.0001.

The animals were monitored for 10 months on various parameters. Circulating levels of GDF15 protein were elevated at 3 months of age and markedly elevated at 6 and 10 months of age (Figure 1A). Because POLG‐mutator animals at 4 months of age with 6 weeks of high‐fat diet feeding previously displayed higher FGF21 circulating levels compared with WT animals under the same conditions (Supporting Information: Reference S38), we measured plasma levels of FGF21 and found elevated circulating FGF21 levels in POLG‐mutator mice at 12 months of age compared with WT animals (Figure 1B). This suggests the development of significant muscle dystrophy at this age, even without additional stress, such as high‐fat diet feeding. Correlating with the marked elevated GDF15 circulating levels, POLG animals displayed significantly stunted body weight gain from around 6 months of age compared with WT animals, which was accompanied by significantly reduced fat mass and fat‐free (lean) mass (Figure 1C,D, Figure S1A). At 10 months of age, we assessed muscle function and exercise capacity of the animals and observed a significant reduction in maximum concentric force of gastrocnemius complex hindlimb muscles and trending reductions in home‐cage voluntary wheel running activities in POLG animals compared with WT control littermates (Figure 1E,F), suggesting impairments of muscle function and a potential exercise intolerance phenotype.

Given the elevated plasma GDF15 levels in POLG animals and the efficacy of an anti‐GDF15 antibody (mAB2) treatment in preserving muscle mass and function in cancer cachexia models from our previous studies [14], we decided to investigate whether GDF15 neutralization with an anti‐GDF15 monoclonal antibody, mAB2, may alleviate muscle atrophy and exercise intolerance in POLG mice.

### 3.2 GDF15 Neutralization Induced Body Weight Gain and Attenuated Lean and Fat Mass Loss in POLG Mutator Mice

**FIGURE 2:**
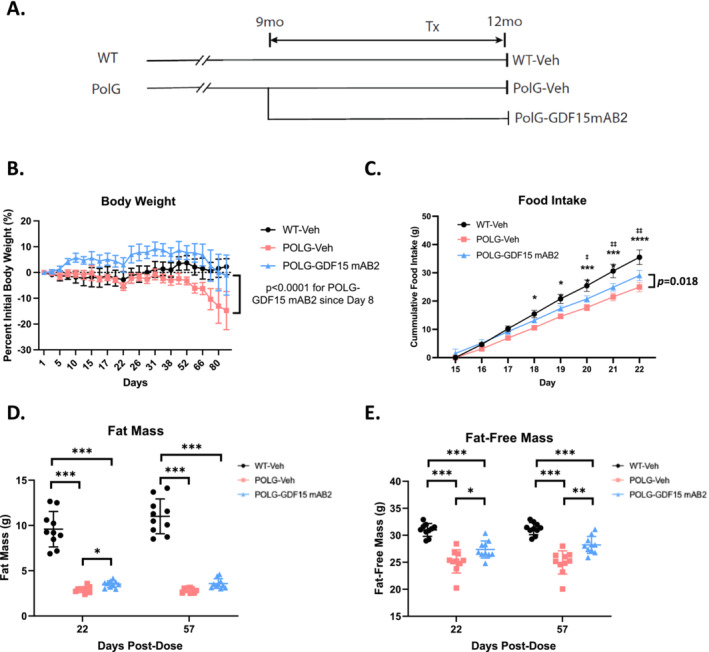
GDF15 neutralization induced body weight gain and attenuates lean and fat mass loss in POLG mutator mice. (A) Schematic representation of the interventional study design to evaluate the effects of an anti‐GDF15 antibody (mAB2) treatment in the POLG model. (B) Percent change in body weight starting from day 1 of the study in WT‐Veh, POLG‐Veh and POLG‐GDF15 mAB2 animals. *n* = 10/group. Significance was assessed by one‐way ANOVA and Tukey's HSD. (C) Cumulative food intake from day 15 after treatment initiation. *n* = 10/group. Significance was assessed by a longitudinal mixed effect model. *p < 0.05, ***p < 0.001, ****p < 0.0001 comparing WT‐Veh and POLG‐Veh. ^‡^ *p* < 0.05, ^‡‡^ *p* < 0.01 comparing WT‐Veh and POLG‐GDF15 mAB2. *p* = 0.018 comparing POLG‐GDF15 mAB2 and POLG‐Veh. (D) Fat mass as measured by EchoMRI at day 22 and 57 post‐initiation of treatment. *n* = 10/group. Significance was assessed by one‐way ANOVA and Tukey's HSD. (E) Fat‐free (lean) mass as measured by EchoMRI at day 22 and 57 post‐initiation of treatment. Significance was assessed by one‐way ANOVA and Tukey's HSD. *n* = 10/group. *p < 0.05, **p < 0.01, ***p < 0.001, ****p < 0.0001.

Based on the longitudinal characterizations of circulating GDF15 levels and changes in muscle function and exercise capacity in POLG mice, we designed an interventional study to assess the effects of the anti‐GDF15 antibody mAB2 as a therapeutic intervention for mitochondria myopathy (Figure 2A). The POLG animals were randomized into two groups based on body weight at the age of 9 months. One group received weekly anti‐GDF15 antibody mAB2 treatment for 12 weeks, whereas the other received weekly control IgG antibody (Vehicle, Veh) treatment. WT control littermates were also included in the study as controls. Strikingly, the anti‐GDF15 antibody treatment induced a significant weight gain in POLG mice after the first week and restored the body weight loss in POLG mice (Figure 2B, Figure S1B,C). This restoration of body weight was accompanied by significantly improved food intake in the POLG‐GDF15 mAB2 group compared with POLG‐Veh animals (Figure 2C), validating that GDF15 neutralization improves appetite and ameliorates the anorexia phenotype in rodents. Body composition measurements, assessed by EchoMRI, revealed that GDF15 neutralization induced trends towards increased fat mass and significant increases in fat‐free (lean) mass at day 22 and 57 post‐treatment (Figure 2D,E), suggesting potential efficacy of the anti‐GDF15 antibody in alleviating muscle atrophy.

### 3.3 GDF15 Neutralization Attenuated Muscle Atrophy in POLG Mutator Mice

**FIGURE 3:**
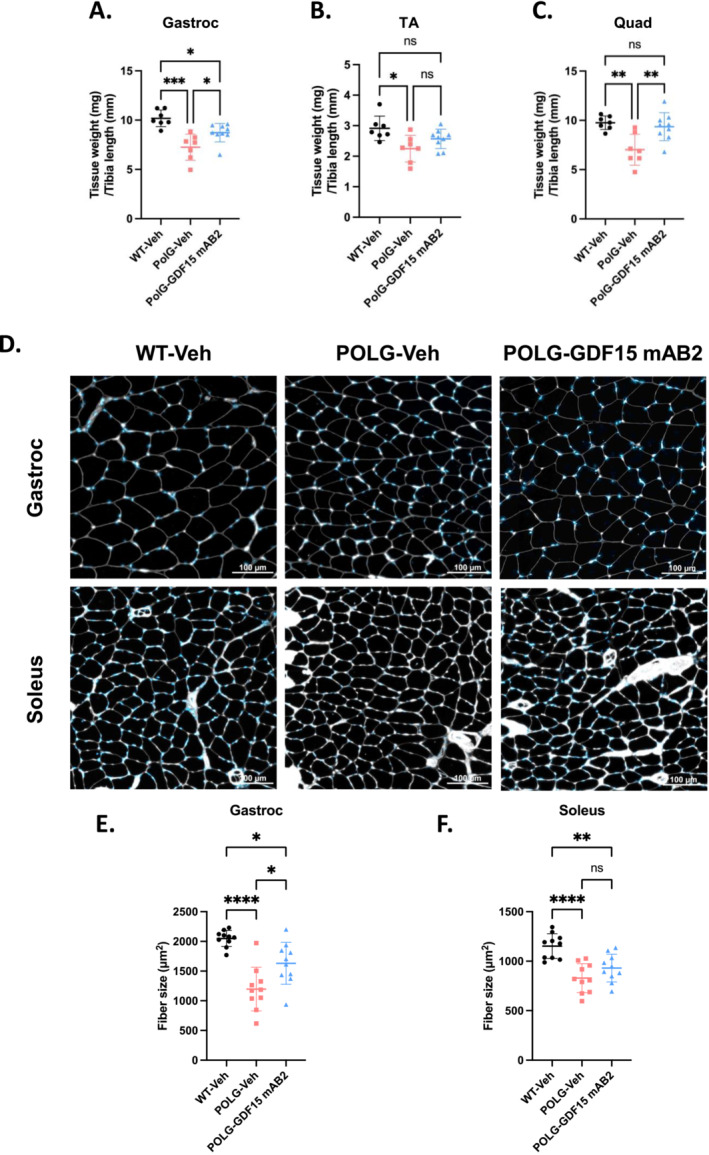
GDF15 neutralization attenuated muscle atrophy in POLG mutator mice. (A) Gastrocnemius (Gastroc) muscle weight normalized to tibia length in WT, POLG‐Veh and POLG‐GDF15 mAB2 animals. *n* = 7 for WT‐veh, *n* = 7 for POG‐veh, *n* = 9 for POLG‐GDF15 mAB2. Significance was assessed by one‐way ANOVA and Tukey's HSD. (B) Tibialis anterior (TA) muscle weight normalized to tibia length in WT, POLG‐Veh and POLG‐GDF15 mAB2 animals. *n* = 7 for WT‐veh, *n* = 7 for POLG‐veh, *n* = 9 for POLG‐GDF15 mAB2. Significance was assessed by one‐way ANOVA and Tukey's HSD. (C) Quadriceps (Quad) muscle weight normalized to tibia length in WT, POLG‐Veh and POLG‐GDF15 mAB2 animals. *n* = 7 for WT‐veh, *n* = 7 for POLG‐veh, *n* = 9 for POLG‐GDF15 mAB2. Significance was assessed by one‐way ANOVA and Tukey's HSD. (D) Representative images of gastrocnemius and soleus muscle sections stained with wheat germ agglutinin (white) and DAPI (blue) for fibre size evaluation. Scale bars = 100 μm. (E) Quantification of gastrocnemius (gastroc) muscle fibre cross‐sectional area (fibre size). *n* = 10/group. Significance was assessed by one‐way ANOVA and Tukey's HSD. (F) Quantification of soleus muscle fibre cross‐sectional area (fibre size). *n* = 10/group. Significance was assessed by one‐way ANOVA and Tukey's HSD. *p < 0.05, **p < 0.01, ***p < 0.001, ****p < 0.0001.

To further characterize the effects of GDF15 neutralization, skeletal muscle was sampled from the animals at the end of the study after 12 weeks of antibody treatment. Reduced gastrocnemius, tibialis anterior and quadriceps muscle mass was observed in POLG animals compared with WT littermates. The anti‐GDF15 antibody treatment led to significant recovery of gastrocnemius and quadriceps muscle mass and a trend towards increased tibialis anterior muscle mass in POLG animals (Figure 3A–C). These results were further validated with fibre size analysis of the gastrocnemius/soleus hindlimb complex using wheat germ agglutinin (white) and DAPI (blue) stain (Figure 3D). Consistent with the muscle weight assessments, a significantly reduced cross‐sectional area of fibres in gastrocnemius and soleus muscle was observed in POLG animals compared with WT littermates. The anti‐GDF15 antibody treatment induced significant increases in cross‐sectional area of fibres in gastrocnemius muscle and, to a lesser degree, in soleus muscle (Figure 3E,F).

### 3.4 GDF15 Neutralization Improved Muscle Function and Exercise Tolerance in POLG Mutator Mice

**FIGURE 4:**
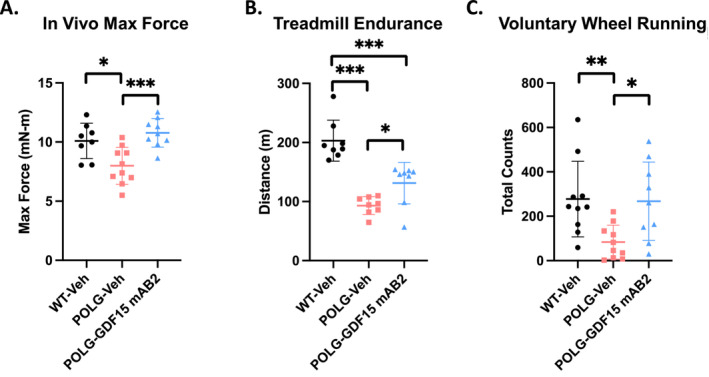
GDF15 neutralization improved skeletal muscle max concentric force and exercise tolerance of POLG mutator mice. (A) In vivo gastrocnemius/soleus complex muscle max concentric force generation in WT‐Veh, POLG‐Veh and POLG‐GDF15 mAB2 animals. *n* = 8 for WT‐Veh, *n* = 10 for PolG‐Veh and *n* = 9 for POLG‐GDF15 mAB2. Significance was assessed by one‐way ANOVA and Tukey's HSD. (B) Distance run until exhaustion during treadmill endurance test. *n* = 8/group. Significance was assessed by one‐way ANOVA and Tukey's HSD. (C) Total wheel turns when given access to running wheels in the home cage. *n* = 10 for WT‐Veh, *n* = 10 for POLG‐Veh and *n* = 9 for POLG‐GDF15 mAB2. Significance was assessed by one‐way ANOVA and Tukey's HSD. *p < 0.05, **p < 0.01, ***p < 0.001.

To investigate whether the increased muscle mass in POLG animals treated with the anti‐GDF15 antibody translated into improved muscle function, muscle max concentric force generation was measured in vivo during electrical stimulation. A reduction in hindlimb muscle max concentric force was observed in POLG animals dosed with IgG control antibody (POLG‐Veh) (Figure 4A). Remarkably, GDF15 neutralization restored the impaired force generation in POLG mice to levels comparable to WT littermate controls (Figure 4A). Additionally, exercise capacity was assessed via treadmill endurance test and voluntary wheel running. Animals from the POLG‐Veh group demonstrated reduced running distance and voluntary wheel running activities, whereas GDF15 neutralization significantly improved these parameters in POLG mice (Figure 4B–C). Collectively, these results support beneficial effects of GDF15 neutralization with a monoclonal antibody on improving muscle function and exercise tolerance in POLG mice.

Exercise capacity and physical performance are known to be affected by both muscle and cardiac function, and cardiac function has previously been shown to be deleteriously affected in POLG mutator mice (Supporting Information: References S39 and S40). To assess whether the anti‐GDF15 antibody treatment affected cardiac function in POLG mice, noninvasive echocardiography was conducted. POLG animals displayed decreased ejection fraction and fractional shortening, along with increased left ventricular end‐diastolic volume, with minimal changes in LV mass (Figure S2A–D), suggesting impaired cardiac function without hypertrophy. Anti‐GDF15 antibody treatment did not improve the impaired cardiac parameters in POLG animals (Figure S2A–D), suggesting that the beneficial effects of GDF15 neutralization on exercise tolerance are not driven by improving cardiac function.

### 3.5 GDF15 Neutralization Reversed the Altered Gene Expression Profile in Gastrocnemius Muscle From POLG Mutator Mice

**FIGURE 5:**
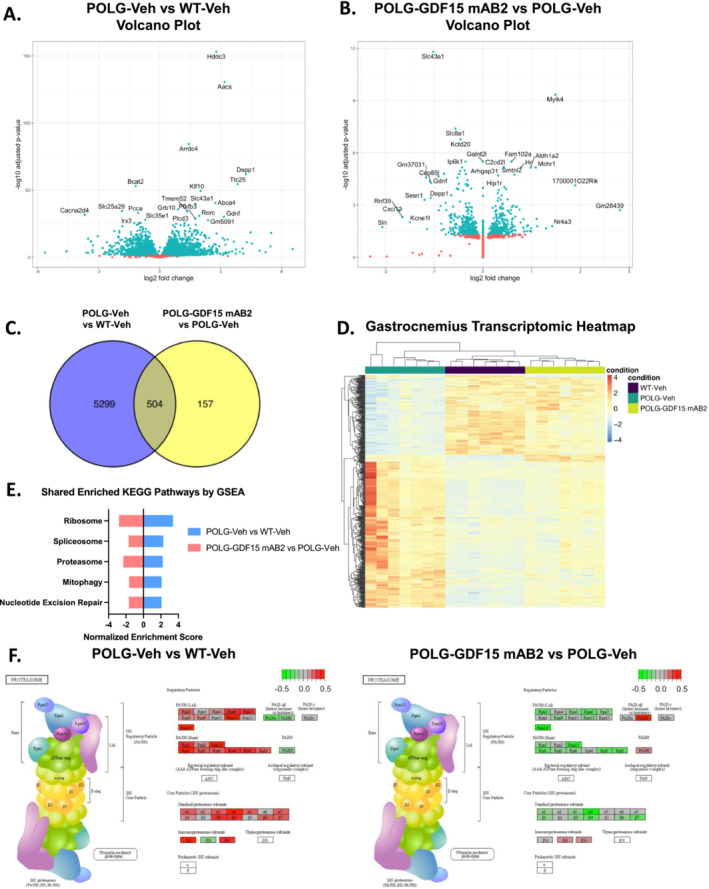
GDF15 neutralization alleviated the abnormal gene expression profile in POLG gastrocnemius muscle. (A) Volcano plot of differentially expressed genes in POLG‐Veh versus WT‐Veh gastrocnemius muscle, following transcriptomic analysis. *n* = 8/group. (B) Volcano plot of differentially expressed genes in POLG‐GDF15 mAB2 versus POLG‐Veh gastrocnemius muscle, following transcriptomic analysis. *n* = 8/group. (C) Venn diagram showing the number of overlapped genes between POLG‐Veh versus WT‐Veh comparison and POLG‐GDF15 mAB2 versus POLG‐Veh comparison. *n* = 8/group. (D) Heatmap of shared differentially expressed genes between POLG‐Veh versus WT‐Veh comparison and POLG‐GDF15 mAB2 versus POLG‐Veh comparison. *n* = 8/group. (E) Top five shared enriched KEGG pathways based on GSEA with directionality of normalized enrichment score in POLG‐Veh versus WT‐Veh and POLG‐GDF15 mAB2 versus POLG‐Veh gastrocnemius muscle. *n* = 8/group. The *p*‐adjusted values for the pathways are ribosome: POLG‐Veh versus WT‐Veh—0.0030, POLG‐GDF15 mAB2 versus POLG‐Veh—0.0026; spliceosome: POLG‐Veh versus WT‐Veh—0.0079, POLG‐GDF15 mAB2 versus POLG‐Veh—0.0026; proteosome: POLG‐Veh versus WT‐Veh—0.0030, POLG‐GDF15 mAB2 versus POLG‐Veh—0.0026; mitophagy: POLG‐Veh versus WT‐Veh—0.0199, POLG‐GDF15 mAB2 versus POLG‐Veh—0.0026; nucleotide excision repair: POLG‐Veh versus WT‐Veh—0.0358, POLG‐GDF15 mAB2 versus POLG‐Veh—0.0026. (F) Graphic representation of genes involved in proteasome signalling and regulation with differences in expression of those genes in POLG‐Veh versus WT‐Veh, as well as in POLG‐GDF15 mAB2 versus POLG‐Veh gastrocnemius muscle. The plots were coloured by the log2 fold changes with max/min colour at 2/−2. Any log2FC greater than 2 or less than −2 received the same max/min colour. *n* = 8/group.

Given the restoration of muscle function and exercise capacity, but not cardiac performance, skeletal muscle was further investigated as a potential site of action for the anti‐GDF15 monoclonal antibody in POLG animals. Gastrocnemius muscle was collected from animals in WT‐Veh, POLG‐Veh and POLG‐GDF15 mAB2 groups at 12 months of age, after 12 weeks of antibody treatment. Transcriptomic analysis (RNAseq) was performed on RNA extracted from these tissues. We observed differential expression of various genes between the POLG‐Veh group and WT‐Veh group, with 2983 genes significantly upregulated, and 2820 genes significantly downregulated in mRNA expression levels (Figure 5A). These data demonstrated that the POLG mutation resulted in a significant perturbation of the skeletal muscle gene expression profile. When comparing the expression profile of muscle from POLG‐GDF15 mAB2 group with the expression profile of muscle from POLG‐Veh group, we found that 278 genes were significantly upregulated, and 383 genes were significantly downregulated (Figure 5B). When we examined the number of overlapping genes that were significantly regulated in both the POLG‐Veh versus WT‐Veh comparison and the POLG‐GDF15 mAB2 versus POLG‐Veh comparison, we identified 504 genes that overlapped between the two comparisons (Figure 5C). Among these 504 genes, the dysregulated expression levels in the POLG‐Veh group compared with the WT‐Veh group were all reversed in the POLG‐GDF15 mAB2 group (Figure 5D). This indicates that anti‐GDF15 mAB2 treatment has beneficial effects in normalizing the transcriptomic profile of gastrocnemius muscle from POLG mice. Additionally, we found that genes related to the citrate cycle and oxidative phosphorylation were downregulated in the POLG‐Veh versus WT‐Veh comparison, indicating impaired mitochondria function, as expected (Figure S3). Notably, anti‐GDF15 mAB2 treatment induced restoration of the expression of genes related to the citrate cycle and oxidative phosphorylation pathways in POLG muscle, suggesting potential benefits of the treatment in improving the mitochondria‐related transcriptomic profile of the muscle. Overall, the analysis indicated that GDF15 neutralization induced a normalization of the gene expression profile of the gastrocnemius muscle from POLG mice, shifting the expression patterns towards the patterns of WT muscle.

For each comparison, GSEA was performed, identifying enriched pathways based on the gene expression profiles. The most enriched KEGG pathways that were significant in both comparisons (POLG‐Veh vs. WT‐Veh and in POLG‐GDF15 mAB2 vs. POLG‐Veh) included the ribosome, spliceosome, proteosome, mitophagy and nucleotide excision repair signalling pathways (Figure 5E). All these pathways were significantly upregulated in POLG‐Veh compared with WT‐Veh and significantly downregulated in POLG‐GDF15 mAB2 compared with POLG‐Veh (Figure 5E), supporting a beneficial effect of the anti‐GDF15 antibody treatment on normalizing the gene expression profile of the gastrocnemius muscle from POLG mice. Proteasomal signalling is of particular interest as muscle atrophy can occur when protein degradation in the muscle outpaces protein synthesis (Supporting Information: Reference S48). Therefore, we took a closer look into the proteasomal signalling pathway in the RNAseq data. A deeper differential gene expression analysis of proteasomal signalling revealed that most proteasome regulatory proteins and core particles are upregulated in POLG‐Veh compared with WT‐Veh and downregulated in POLG‐GDF15 mAB2 compared with POLG‐Veh (Figure 5F). This indicates that GDF15 neutralization may exhibit its beneficial effects on restoring muscle atrophy in POLG mice through normalizing proteasomal signalling.

### 3.6 GDF15 Neutralization Reduced Circulating Corticosterone Levels and Suppressed Glucocorticoid Target Gene Expression in Muscle of POLG Mutator Mice

**FIGURE 6:**
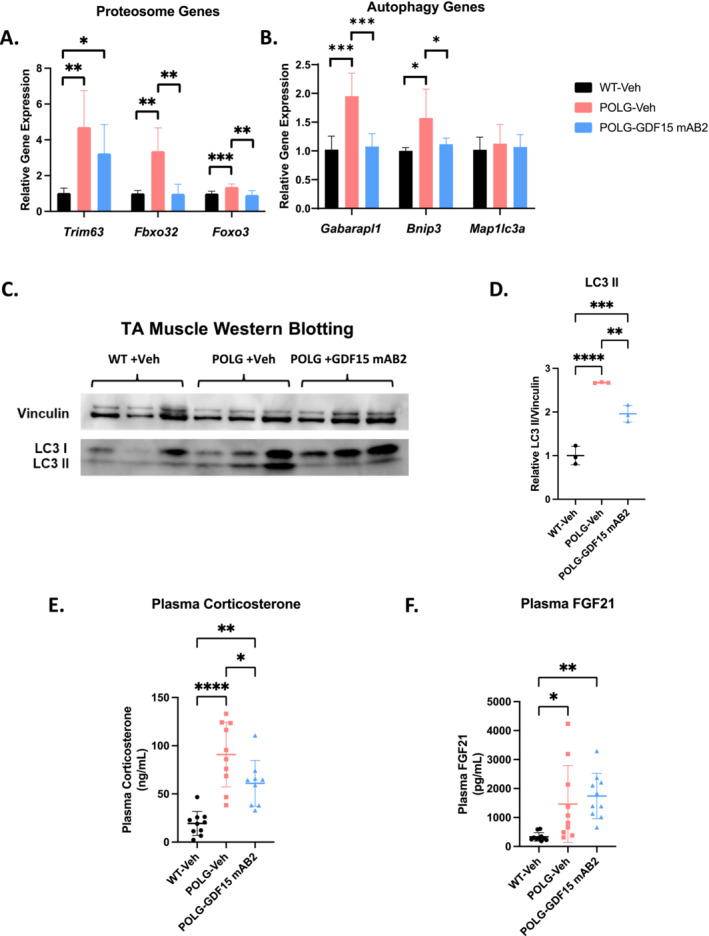
GDF15 neutralization reduced circulating corticosterone levels and suppressed glucocorticoid target gene expression in the tibialis anterior muscle of POLG mice. (A) Gene expression of genes involved in proteasome signalling in tibialis anterior muscle, assessed by qPCR. *n* = 7/group. Significance was assessed by one‐way ANOVA and Tukey's HSD. (B) Gene expression of genes involved in autophagy signalling in tibialis anterior muscle, assessed by qPCR. *n* = 7/group. Significance was assessed by one‐way ANOVA and Tukey's HSD. (C) Western blot images of Vinculin and LC3 in tibialis anterior muscle. *n* = 3/group. (D) Quantification of Figure 6C. *n* = 3/group. Significance was assessed by one‐way ANOVA and Tukey's HSD. (E) Plasma corticosterone levels at day 87 post‐initiation of treatment. *n* = 10 for WT‐Veh and POLG‐Veh and *n* = 9 for POLG‐GDF15 mAB2. Significance was assessed by one‐way ANOVA and Tukey's HSD. (F) Plasma FGF21 levels at day 87 post‐initiation of treatment. *n* = 10/group. The source data for WT‐Veh and POLG‐Veh groups in Figure 6F is the same as Figure 1B. Significance was assessed by one‐way ANOVA and Tukey's HSD. *p < 0.05, **p < 0.01, ***p < 0.001, ****p < 0.0001.

To investigate whether the expression changes in genes involved in proteasomal signalling revealed by RNAseq analysis of gastrocnemius muscle are conserved in another muscle with similar fibre compositions, RT‐qPCR was conducted for proteasomal signalling gene hits from the RNAseq analysis using tibialis anterior muscle. The same expression pattern of these genes was found in tibialis anterior muscle: The expression of *Trim63*, *Fbxo32* and *Foxo3* was increased in the POLG‐Veh group compared with the WT‐Veh group and GDF15 neutralization induced reductions in the expression of these genes (Figure 6A). The same observations were made for genes involved in autophagic signalling: we found increases in *Gabarapl1* and *Bnip3* gene expression in the POLG‐Veh group compared with the WT‐Veh group and anti‐GDF15 antibody treatment reversed the changes (Figure 6B). Although *Map 1lc3a* (LC3) gene expression was not altered between the groups (Figure 6B), LC3II protein levels were significantly increased in POLG‐Veh muscle and GDF15 neutralization induced a significant reduction in LC3II protein levels in POLG muscle, suggesting a potential restoration of the autophagy pathway (Figure 6C,D).

Further, when we looked at the differentially expressed genes as a whole, many of the genes with altered expression are downstream targets of the glucocorticoid receptor in muscle [20], suggesting a potential role of the glucocorticoid pathway in mediating the effects of GDF15 neutralization. It was previously reported that circulating corticosterone levels are affected by GDF15, and the glucocorticoid pathway mediates some of the downstream effects of GDF15 neutralization [14, 21]. Therefore, we measured the plasma corticosterone levels and found that plasma corticosterone levels were significantly increased in POLG animals and that elevation was reduced following anti‐GDF15 antibody treatment (Figure 6E). Because deletion of FGF21 in OPA1‐deficient mice (a model of mitochondrial myopathy) resulted in partial attenuation of muscle wasting and prolonged survival [10], and the findings that FGF21 increased circulating glucocorticoid levels and suppressed physical activity [22], we investigated whether GDF15 neutralization reduced FGF21 levels, leading to improvements of disease conditions as well as reductions in circulating corticosterone levels. We found that the elevated circulating levels of FGF21 were not significantly reduced by GDF15 neutralization in POLG animals (Figure 6F), excluding FGF21 from being the downstream mediator of the beneficial effects upon GDF15 neutralization.

## 4 Discussion

Primary mitochondrial myopathy (PMM) is a debilitating disease caused by mutations in mtDNA or nDNA‐encoded genes of mitochondrial proteins that predominantly, but not exclusively, affect skeletal muscle. The most common symptoms are muscle weakness, exercise intolerance and progressive external ophthalmoplegia [1, 17, 23]. Interestingly, elevated circulating GDF15 levels were reported in patients with mitochondrial diseases, including PMM; thus, it has been put forward as a useful biomarker for mitochondrial disorders [23, 24] (Supporting Information: Reference S41), as well as a potential therapeutic biomarker for myopathy in patients with thymidine kinase 2 (TK2) deficiency [1, 25]. Currently, there is no disease‐defining treatment available for patients of PMM.

Previous publications indicate a causal role of GDF15 in tumour‐induced anorexia, weight loss, muscle function decline and exercise intolerance [16]. Pharmacological administration of GDF15 protein reduced voluntary wheel running activity, whereas neutralization of GDF15 with an anti‐GDF15 antibody improved exercise performance, indicating a role for GDF15 in regulating muscle function, physical activity and exercise performance [14, 26]. Notably, a recent phase 2 clinical study demonstrated that ponsegromab, an anti‐GDF15 antibody, induced weight gain, improved physical activity and reduced cachexia symptoms in patients with cancer cachexia, confirming GDF15's role as a key driver of cachexia (Supporting Information: Reference S26). Elevated circulating levels of GDF15 have been reported in patients with mitochondrial myopathies. However, it remains unknown whether GDF15 contributes to muscle weakness, fatigue and exercise intolerance in mitochondrial myopathy and whether GDF15 blockade could be a potential therapy for PMM. Thus, this study aimed to investigate the effects and potential mechanisms of GDF15 neutralization on muscle function and physical performance in the Polg^D257A/D257A^ mtDNA mutator mice (POLG mice), a preclinical model displaying many features of PMM patients. The results may provide insights into the potential therapeutic application of GDF15 blockade to improve muscle function, exercise intolerance, physical ability and ultimately the quality of life of patients with mitochondrial disorders.

Consistent with previously published data [19, 27], we observed that the POLG mice displayed body weight loss, skeletal muscle atrophy, impaired muscle function and exercise intolerance, which manifested as early as 6 months of age when compared with WT littermate controls (Figures 1 and 2). These impairments were accompanied by elevated circulating levels of both GDF15 and FGF21 (Figure 1A,B). The presented abnormalities in the POLG mice are reminiscent of the symptoms of PMM in patients, which prompted us to examine the effects of GDF15 neutralization in these mice with a selective and potent monoclonal anti‐GDF15 antibody (mAB2), which has demonstrated robust efficacy in reversing tumour‐ or cisplatin‐induced cachexia in our previous studies [14, 16]. Given the prominent role of GDF15 in regulating appetite and body weight, we first evaluated the effect of GDF15 neutralization on these parameters. The treatment was initiated at 9 months of age when the disease conditions in POLG animals manifested as a therapeutic intervention. The POLG mice treated with the anti‐GDF15 antibody gained more weight and consumed more food when compared with the vehicle control group during the 12‐week treatment (Figure 2B,C), further validating the beneficial effects of GDF15 neutralization on appetite control and body weight regulation. The fat‐free lean mass is also significantly higher in the anti‐GDF15 antibody‐treated POLG mice compared with the vehicle control group, indicating a restoration of muscle atrophy. With the demonstrated efficacy of the anti‐GDF15 antibody on these parameters, we further assessed the effects of GDF15 neutralization on muscle function and exercise capacity. The POLG mice displayed reduced hindlimb skeletal muscle (gastrocnemius, tibialis anterior and quadriceps) mass and fibre size, and treatment with anti‐GDF15 antibody significantly attenuated muscle atrophy (Figure 3). More importantly, the anti‐GDF15 antibody treatment completely restored muscle function, as assessed by max concentric force generation (Figure 4A). Moreover, we found that POLG mice demonstrated reduced voluntary wheel running activity and physical performance on forced treadmill running. The anti‐GDF15 antibody treatment significantly enhanced voluntary wheel running activity as well as treadmill endurance in POLG mice (Figure 4B,C), indicating that GDF15 neutralization improved not only muscle mass and function but also physical performance, which is a critical parameter associated with immobility and quality of life in patients with PMM.

In our previous study investigating the effects of GDF15 neutralization in a mouse model of cancer cachexia [14], we found that the beneficial effects on muscle function and physical performance are primarily attributed to increased caloric intake. Consistent with these findings, we also observed significantly increased food intake in the POLG mice upon anti‐GDF15 antibody treatment, accompanied by improved muscle function and physical performance. It is highly likely that the increased caloric intake induced by the treatment contributed to the improved body weights, muscle function and physical performance in the POLG mice, though additional and alternative mechanisms could also play a role.

To gain more insights into the mechanisms underlying the beneficial effects of the anti‐GDF15 antibody treatment, gastrocnemius muscle RNA‐seq profiling was performed. GDF15 neutralization demonstrated significant beneficial effects in normalizing the transcriptomic profile of gastrocnemius muscle from POLG mice (Figure 5A–D). Additionally, GSEA analysis revealed that the most enriched KEGG pathways upregulated by the POLG mutations and downregulated by the anti‐GDF15 antibody treatment include ribosome, spliceosome, proteosome, mitophagy and nucleotide excision repair signalling pathways (Figure 5E). Dysregulation of proteasomal signalling and mitophagy often contributes to muscle atrophy [8] (Supporting Information: References S42–S44), which occurrs when protein degradation outpaces protein synthesis. Increased protein degradation may occur through the activation of the UPS and/or mitophagy/autophagy/lysosome pathways [28]. These pathways are activated coordinately by transcription factors of the Foxo family [29, 30, 31] and NF‐kappaB family [32, 33]. Among the Foxo family members, Foxo3 is the most critical factor in regulating these pathways contributing to muscle atrophy [31, 34]. We found that *Foxo3*, but not *Foxo1* or *Foxo4*, was significantly upregulated in muscles from POLG mice and this upregulation was normalized by anti‐GDF15 antibody treatment (Figure 5, Figure 6A). Additionally, the expression of E3 ubiquitin ligases, *Trim63/MuRF1* and *Fbxo32/MAFBX* (known downstream targets of Foxo3 and crucial regulators of muscle degradation) [35, 36], was also upregulated in muscles from POLG mice and suppressed by anti‐GDF15 antibody treatment (Figure 5, Figure 6A). *Depp1*, one of most significantly upregulated genes in POLG muscle and downregulated with anti‐GDF15 antibody treatment (Figure 5A,B), is a known target gene of *Foxo3* and a critical mediator of Foxo3‐mediated autophagy [37]. Furthermore, LC3II protein levels increased significantly in POLG muscle and the change was restored with anti‐GDF15 antibody treatment (Figure 6C,D). These data collectively suggest that the POLG muscle demonstrated dysregulated proteasomal signalling and autophagy and that GDF15 neutralization may exert its beneficial effects in part by restoring these abnormalities. Recent publications have shown that increases in gene expression and protein levels of autophagic markers could be detected without significant changes in autophagic flux (Supporting Information: References S49–S52). Future studies are needed to evaluate potential changes in autophagic flux and to further elucidate the potential mechanisms underlying GDF15 neutralization on autophagy and mitophagy.

Glucocorticoids (e.g., cortisol), and their synthetic analogue dexamethasone, are known to trigger muscle atrophy [38, 39]. Released from the adrenal gland in stressful conditions, these steroids are essential for muscle wasting during fasting, renal failure, diabetes sepsis and cancer, by inhibiting protein synthesis and promoting proteolysis [40] (Supporting Information: References S45 and S46). GDF15 has been reported to potently activate the hypothalamic–pituitary–adrenal (HPA) axis, increasing circulating corticosterone levels in mice and rats [21]. Therefore, it is conceivable that the elevated GDF15 levels observed in POLG mice may activate the HPA axis and raise corticosterone levels, which, in turn, would trigger transcriptional activation of muscle atrophy programs, impairing muscle function and physical performance. Indeed, plasma corticosterone levels were significantly elevated in POLG mice compared with WT littermates, and this elevation was significantly attenuated by anti‐GDF15 antibody treatment (Figure 6E). This suggests that suppression of corticosterone levels and the glucocorticoid pathway could be a therapeutic mechanism for GDF15 neutralization on alleviating muscle wasting and impaired physical activity in POLG animals. Besides corticosterone, other factors and pathways, such as inflammatory signalling and growth factors, likely play important roles in the pathogenesis of mitochondrial myopathy and mediate the beneficial effects of GDF15 neutralization in POLG mice. Further experiments using glucocorticoid antagonists are required to confirm that the anti‐atrophic effect of GDF15 neutralization is corticosterone‐driven. Additional investigations are also needed to profile the full range of downstream signalling and mechanisms underlying GDF15 neutralization in POLG model.

FGF21 has been studied in the context of POLG‐mutator animals and has been shown to act primarily in the adipose tissues (Supporting Information: Reference S38). Elevated FGF21 levels have been reported in patients and preclinical models with mitochondrial disorders [1, 9]. Deletion of FGF21 in OPA1‐deficient mice, a model of mitochondrial myopathy, resulted in partial attenuation of muscle wasting and prolonged survival [10]. Interestingly, FGF21 was also reported to increase circulating glucocorticoid levels and suppress physical activity by acting through β‐Klotho, which is expressed in the suprachiasmatic nucleus of the hypothalamus and the dorsal vagal complex of the hindbrain [22]. These data suggest that FGF21 has a causal role in muscle wasting and impairment of physical function in mitochondrial myopathy. In our study, the POLG animals at 12 months of age demonstrated a significant elevation in circulating levels of FGF21 and these elevations were not restored with GDF15 neutralization (Figure 6F). These findings indicate a potential selective regulation of the glucocorticoid pathway by GDF15 neutralization and suggest that a combination of both GDF15 and FGF21 signalling inhibition could provide greater efficacy in alleviating mitochondrial myopathy.

There are several limitations of our current study. First, although we found that GDF15 mAB2 treatment restored transcriptional perturbation in multiple pathways in muscle from the POLG mice, future studies with direct measurement of mitochondrial function, mitochondrial dynamics and quality and protein turnover are needed to further define the mechanisms mediating GDF15 neutralization's effects on skeletal muscle mass and function in this model. Second, POLG mutator mice are known to develop an anaemic phenotype and GDF15 has been implicated in regulating erythroid homeostasis (Supporting Information: References S52 and S53). Anaemia plays a crucial role in regulating skeletal muscle function (Supporting Information: Reference S54), thus it is important to investigate the effects of GDF15 neutralization on the anaemia phenotype in POLG mutator mice in future studies. These additional experiments and data will provide more mechanistic insights into the therapeutic mechanisms of GDF15 neutralization for mitochondrial myopathy and are warranted for future investigations.

**FIGURE 7:**
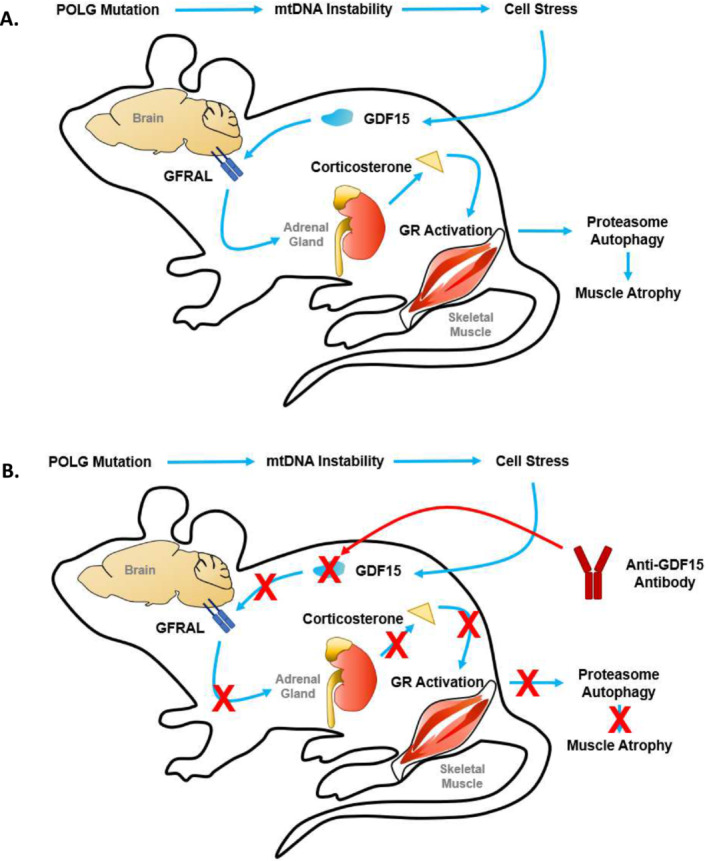
Graphical model of potential mechanisms underlying the beneficial effects of GDF15 neutralization on POLG mice. (A) The POLG mutation decreases mtDNA stability while increasing circulating levels of GDF15 and corticosterone. This leads to increased proteasomal/autophagic signalling in the skeletal muscle, decreased fat‐free mass and impaired muscle function. (B) Treatment with anti‐GDF15 antibody mAB2 neutralizes circulating GDF15, restores proteasomal/autophagic signalling in the skeletal muscle and improves fat‐free mass and muscle function.

In summary, our results demonstrated that GDF15 neutralization ameliorated body weight loss, attenuated muscle atrophy and improved muscle function and physical performance in a rodent model of mitochondrial myopathy (POLG). Beyond appetite control and increased caloric intake, one of the potential mechanisms underlying the beneficial effects of GDF15 neutralization, as depicted in Figure 7, involves the suppression of circulating levels of corticosterone and the activation of downstream signalling pathways such as the mitophagy/autophagy and proteasome signalling pathways (Figure 7). Collectively, the results of the study highlight the potential for GDF15 neutralization with a monoclonal antibody as a therapeutic avenue to improve physical performance and mitigate adverse clinical outcomes for patients with PMM or similar disorders.

## Conflicts of Interest

SEF, LS, AB, AR, AS, BZ and ZW were employees of Pfizer when the study was conducted. All other authors are employees and shareholders of Pfizer.

## Supporting information


**Figure S1** GDF15 Neutralization Induced Body weight Gain in POLG mutator mice.
**Figure S2.** GDF15 Neutralization Does Not Affect Cardiac Function in POLG Mutator Mice.
**Figure S3.** GDF15 Neutralization Alleviated Altered Gene Expression Profile of Citrate Cycle Genes and Oxidative Phosphorylation Genes in POLG Gastrocnemius Muscle.

**Data S1** Supplementary References.

## References

[1] G. S. Gorman , A. M. Schaefer , Y. Ng , et al., “Prevalence of Nuclear and Mitochondrial DNA Mutations Related to Adult Mitochondrial Disease,” Annals of Neurology 77, no. 5 (2015): 753–759. 25652200 10.1002/ana.24362 PMC4737121

[2] S. T. Ahmed , L. Craven , O. M. Russell , D. M. Turnbull , and A. E. Vincent , “Diagnosis and Treatment of Mitochondrial Myopathies,” Neurotherapeutics 15, no. 4 (2018): 943–953. 30406383 10.1007/s13311-018-00674-4 PMC6277287

[3] I. P. de Barcelos , V. Emmanuele , and M. Hirano , “Advances in Primary Mitochondrial Myopathies,” Current Opinion in Neurology 32, no. 5 (2019): 715–721. 31408013 10.1097/WCO.0000000000000743 PMC6938233

[4] C. Wang and R. J. Youle , “The Role of Mitochondria in Apoptosis*,” Annual Review of Genetics 43 (2009): 95–118. 10.1146/annurev-genet-102108-134850 PMC4762029 19659442

[5] M. Khaidakov , R. H. Heflich , M. G. Manjanatha , M. B. Myers , and A. Aidoo , “Accumulation of Point Mutations in Mitochondrial DNA of Aging Mice,” Mutation Research 526, no. 1–2 (2003): 1–7. 12714177 10.1016/s0027-5107(03)00010-1

[6] S. R. Schwarze , C. M. Lee , S. S. Chung , E. B. Roecker , R. Weindruch , and J. M. Aiken , “High Levels of Mitochondrial DNA Deletions in Skeletal Muscle of Old Rhesus Monkeys,” Mechanisms of Ageing and Development 83, no. 2 (1995): 91–101. 8569289 10.1016/0047-6374(95)01611-3

[7] S. C. Zapico and D. H. Ubelaker , “mtDNA Mutations and Their Role in Aging, Diseases and Forensic Sciences,” Aging and Disease 4, no. 6 (2013): 364–380. 24307969 10.14336/AD.2013.0400364 PMC3843653

[8] G. B. Kubat , E. Bouhamida , O. Ulger , et al., “Mitochondrial Dysfunction and Skeletal Muscle Atrophy: Causes, Mechanisms, and Treatment Strategies,” Mitochondrion 72 (2023): 33–58. 37451353 10.1016/j.mito.2023.07.003

[9] C. E. Wall , J. Whyte , J. M. Suh , et al., “High‐Fat Diet and FGF21 Cooperatively Promote Aerobic Thermogenesis in mtDNA Mutator Mice,” Proceedings of the National Academy of Sciences of the United States of America 112, no. 28 (2015): 8714–8719. 26124126 10.1073/pnas.1509930112 PMC4507233

[10] C. Tezze , V. Romanello , M. A. Desbats , et al., “Age‐Associated Loss of OPA1 in Muscle Impacts Muscle Mass, Metabolic Homeostasis, Systemic Inflammation, and Epithelial Senescence,” Cell Metabolism 25, no. 6 (2017): 1374–1389 e6. 28552492 10.1016/j.cmet.2017.04.021 PMC5462533

[11] D. Wang , E. A. Day , L. K. Townsend , D. Djordjevic , S. B. Jørgensen , and G. R. Steinberg , “GDF15: Emerging Biology and Therapeutic Applications for Obesity and Cardiometabolic Disease,” Nature Reviews. Endocrinology 17, no. 10 (2021): 592–607. 10.1038/s41574-021-00529-7 34381196

[12] S. E. Mullican , X. Lin‐Schmidt , C. N. Chin , et al., “GFRAL Is the Receptor for GDF15 and the Ligand Promotes Weight Loss in Mice and Nonhuman Primates,” Nature Medicine 23, no. 10 (2017): 1150–1157. 10.1038/nm.4392 28846097

[13] I. S. Anand , T. Kempf , T. S. Rector , et al., “Serial Measurement of Growth‐Differentiation Factor‐15 in Heart Failure: Relation to Disease Severity and Prognosis in the Valsartan Heart Failure Trial,” Circulation 122, no. 14 (2010): 1387–1395. 20855664 10.1161/CIRCULATIONAHA.109.928846

[14] J. Y. Kim‐Muller , L. J. Song , B. LaCarubba Paulhus , et al., “GDF15 Neutralization Restores Muscle Function and Physical Performance in a Mouse Model of Cancer Cachexia,” Cell Reports 42, no. 1 (2023): 111947. 36640326 10.1016/j.celrep.2022.111947

[15] D. M. Breen , S. Jagarlapudi , A. Patel , et al., “Growth Differentiation Factor 15 Neutralization Does Not Impact Anorexia or Survival in Lipopolysaccharide‐Induced Inflammation,” iScience 24, no. 6 (2021): 102554. 34189431 10.1016/j.isci.2021.102554 PMC8215224

[16] D. M. Breen , H. Kim , D. Bennett , et al., “GDF‐15 Neutralization Alleviates Platinum‐Based Chemotherapy‐Induced Emesis, Anorexia, and Weight Loss in Mice and Nonhuman Primates,” Cell Metabolism 32, no. 6 (2020): 938–950. 33207247 10.1016/j.cmet.2020.10.023

[17] M. Mancuso , R. McFarland , T. Klopstock , et al., “International Workshop:: Outcome Measures and Clinical Trial Readiness in Primary Mitochondrial Myopathies in Children and Adults. Consensus Recommendations. 16–18 November 2016, Rome, Italy,” Neuromuscular Disorders 27, no. 12 (2017): 1126–1137. 29074296 10.1016/j.nmd.2017.08.006 PMC6094160

[18] S. Rahman and W. C. Copeland , “POLG‐Related Disorders and Their Neurological Manifestations,” Nature Reviews. Neurology 15, no. 1 (2019): 40–52. 30451971 10.1038/s41582-018-0101-0 PMC8796686

[19] A. Trifunovic , A. Wredenberg , M. Falkenberg , et al., “Premature Ageing in Mice Expressing Defective Mitochondrial DNA Polymerase,” Nature 429, no. 6990 (2004): 417–423. 15164064 10.1038/nature02517

[20] M. K. Lee , H. H. Jeong , M. J. Kim , H. Ryu , J. Baek , and B. Lee , “Nutrients Against Glucocorticoid‐Induced Muscle Atrophy,” Food 11, no. 5 (2022): 687. 10.3390/foods11050687 PMC8909279 35267320

[21] I. Cimino , H. Kim , Y. C. L. Tung , et al., “Activation of the Hypothalamic‐Pituitary‐Adrenal Axis by Exogenous and Endogenous GDF15,” Proceedings of the National Academy of Sciences of the United States of America 118, no. 27 (2021): e2106868118. 34187898 10.1073/pnas.2106868118 PMC8271778

[22] A. L. Bookout , M. H. M. de Groot , B. M. Owen , et al., “FGF21 Regulates Metabolism and Circadian Behavior by Acting on the Nervous System,” Nature Medicine 19, no. 9 (2013): 1147–1152. 10.1038/nm.3249 PMC3769420 23933984

[23] V. Montano , F. Gruosso , V. Carelli , et al., “Primary Mitochondrial Myopathy: Clinical Features and Outcome Measures in 118 Cases From Italy,” Neurology Genetics 6, no. 6 (2020): e519. 33209982 10.1212/NXG.0000000000000519 PMC7670572

[24] Y. Li , S. Li , Y. Qiu , et al., “Circulating FGF21 and GDF15 as Biomarkers for Screening, Diagnosis, and Severity Assessment of Primary Mitochondrial Disorders in Children,” Frontiers in Pediatrics 10 (2022): 851534. 35498801 10.3389/fped.2022.851534 PMC9047692

[25] C. Dominguez‐Gonzalez , C. Badosa , M. Madruga‐Garrido , et al., “Growth Differentiation Factor 15 Is a Potential Biomarker of Therapeutic Response for TK2 Deficient Myopathy,” Scientific Reports 10, no. 1 (2020): 10111. 32572108 10.1038/s41598-020-66940-8 PMC7308386

[26] A. B. Klein , T. S. Nicolaisen , N. Ørtenblad , et al., “Pharmacological but Not Physiological GDF15 Suppresses Feeding and the Motivation to Exercise,” Nature Communications 12, no. 1 (2021): 1041. 10.1038/s41467-021-21309-x PMC7884842 33589633

[27] A. Safdar , J. M. Bourgeois , D. I. Ogborn , et al., “Endurance Exercise Rescues Progeroid Aging and Induces Systemic Mitochondrial Rejuvenation in mtDNA Mutator Mice,” Proceedings of the National Academy of Sciences of the United States of America 108, no. 10 (2011): 4135–4140. 21368114 10.1073/pnas.1019581108 PMC3053975

[28] R. Piccirillo , F. Demontis , N. Perrimon , and A. L. Goldberg , “Mechanisms of Muscle Growth and Atrophy in Mammals and Drosophila,” Developmental Dynamics 243, no. 2 (2014): 201–215. 24038488 10.1002/dvdy.24036 PMC3980484

[29] D. J. Glass , “Signaling Pathways Perturbing Muscle Mass,” Current Opinion in Clinical Nutrition and Metabolic Care 13, no. 3 (2010): 225–229. 20397318 10.1097/mco.0b013e32833862df

[30] M. Sandri , C. Sandri , A. Gilbert , et al., “Foxo Transcription Factors Induce the Atrophy‐Related Ubiquitin Ligase Atrogin‐1 and Cause Skeletal Muscle Atrophy,” Cell 117, no. 3 (2004): 399–412. 15109499 10.1016/s0092-8674(04)00400-3 PMC3619734

[31] J. Zhao , J. J. Brault , A. Schild , et al., “FoxO3 Coordinately Activates Protein Degradation by the Autophagic/Lysosomal and Proteasomal Pathways in Atrophying Muscle Cells,” Cell Metabolism 6, no. 6 (2007): 472–483. 18054316 10.1016/j.cmet.2007.11.004

[32] D. Cai , J. D. Frantz , N. E. Tawa, Jr. , et al., “IKKbeta/NF‐kappaB Activation Causes Severe Muscle Wasting in Mice,” Cell 119, no. 2 (2004): 285–298. 15479644 10.1016/j.cell.2004.09.027

[33] R. B. Hunter , E. J. Stevenson , A. Koncarevic , H. Mitchell‐Felton , D. A. Essig , and S. C. Kandarian , “Activation of an Alternative NF‐κB Pathway in Skeletal Muscle During Disuse Atrophy,” FASEB Journal 16, no. 6 (2002): 529–538. 11919155 10.1096/fj.01-0866com

[34] G. Milan , V. Romanello , F. Pescatore , et al., “Regulation of Autophagy and the Ubiquitin‐Proteasome System by the FoxO Transcriptional Network During Muscle Atrophy,” Nature Communications 6 (2015): 6670. 10.1038/ncomms7670 PMC4403316 25858807

[35] M. D. Gomes , S. H. Lecker , R. T. Jagoe , A. Navon , and A. L. Goldberg , “Atrogin‐1, a Muscle‐Specific F‐Box Protein Highly Expressed During Muscle Atrophy,” Proceedings of the National Academy of Sciences of the United States of America 98, no. 25 (2001): 14440–14445. 11717410 10.1073/pnas.251541198 PMC64700

[36] S. C. Bodine , E. Latres , S. Baumhueter , et al., “Identification of Ubiquitin Ligases Required for Skeletal Muscle Atrophy,” Science 294, no. 5547 (2001): 1704–1708. 11679633 10.1126/science.1065874

[37] S. Salcher , M. Hermann , U. Kiechl‐Kohlendorfer , M. J. Ausserlechner , and P. Obexer , “C10ORF10/DEPP‐Mediated ROS Accumulation Is a Critical Modulator of FOXO3‐Induced Autophagy,” Molecular Cancer 16, no. 1 (2017): 95. 28545464 10.1186/s12943-017-0661-4 PMC5445297

[38] B. Dahlmann , M. Rutschmann , and H. Reinauer , “Effect of Starvation or Treatment With Corticosterone on the Amount of Easily Releasable Myofilaments in Rat Skeletal Muscles,” Biochemical Journal 234, no. 3 (1986): 659–664. 3718490 10.1042/bj2340659 PMC1146622

[39] A. L. Goldberg and H. M. Goodman , “Relationship Between Cortisone and Muscle Work in Determining Muscle Size,” Journal of Physiology 200, no. 3 (1969): 667–675. 5765854 10.1113/jphysiol.1969.sp008715 PMC1350520

[40] T. P. Braun and D. L. Marks , “The Regulation of Muscle Mass by Endogenous Glucocorticoids,” Frontiers in Physiology 6 (2015): 12. 25691871 10.3389/fphys.2015.00012 PMC4315033

