# Supplementary material for: GDF15 Neutralization Ameliorates Muscle Atrophy and Exercise Intolerance in a Mouse Model of Mitochondrial Myopathy
Source: J Cachexia Sarcopenia Muscle. 2025 Feb 20;16(1):e13715. doi: 10.1002/jcsm.13715 (PMC11840706; doi:10.1002/jcsm.13715)
Supplement: Supplementary file 1 — Figure S1 GDF15 Neutralization Induced Body weight Gain in POLG mutator mice. Figure S2. GDF15 Neutralization Does Not Affect Cardiac Function in POLG Mutator Mice. Figure S3. GDF15 Neutralization Alleviated Altered Gene Expression Profile of Citrate Cycle Genes and Oxidative Phosphorylation Genes in POLG Gastrocnemius Muscle. [file JCSM-16-e13715-s002.pptx]

## Slide 1
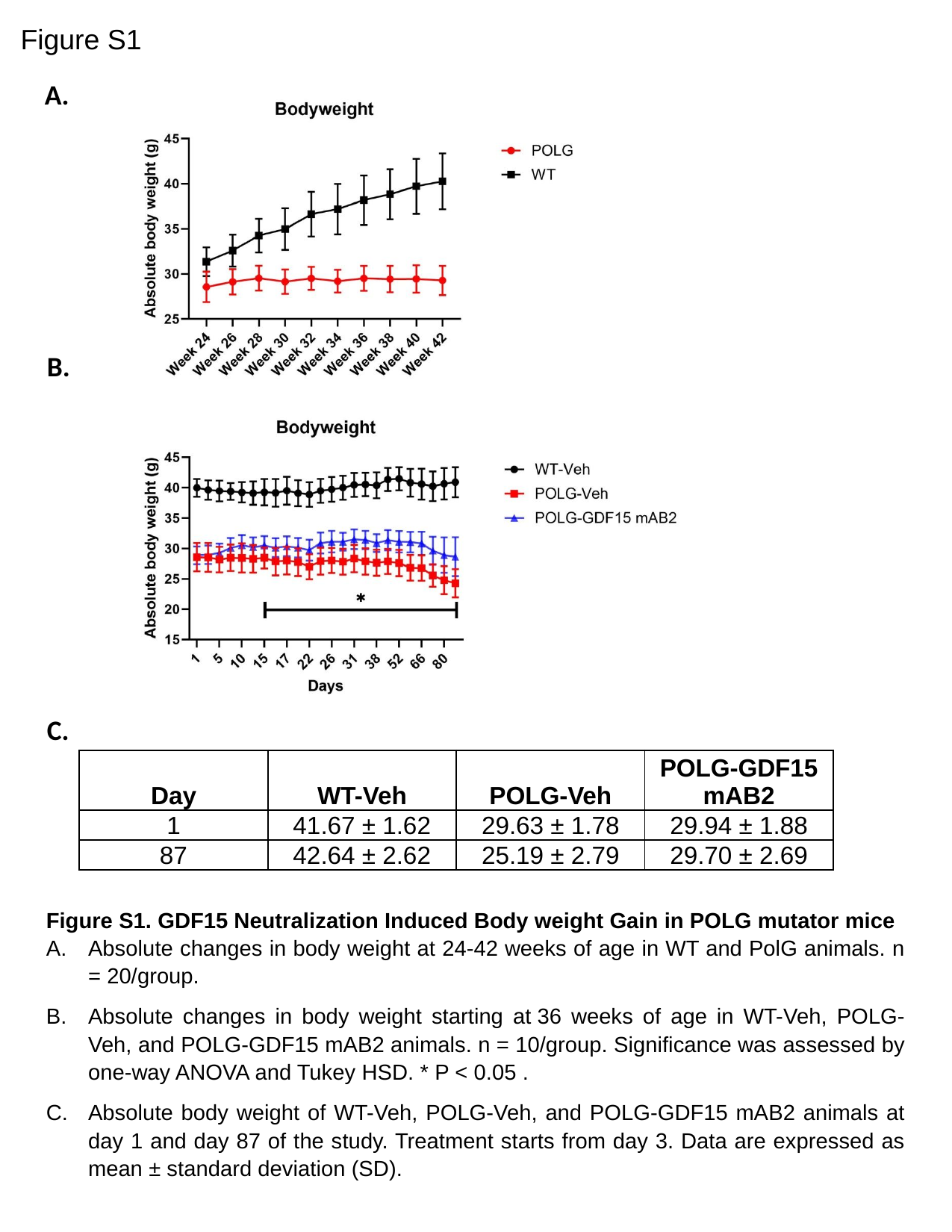

Figure S1
A.
B.
C.
| Day | WT-Veh | POLG-Veh | POLG-GDF15 mAB2 |
| --- | --- | --- | --- |
| 1 | 41.67 ± 1.62 | 29.63 ± 1.78 | 29.94 ± 1.88 |
| 87 | 42.64 ± 2.62 | 25.19 ± 2.79 | 29.70 ± 2.69 |
Figure S1. GDF15 Neutralization Induced Body weight Gain in POLG mutator mice
Absolute changes in body weight at 24-42 weeks of age in WT and PolG animals. n = 20/group.
Absolute changes in body weight starting at 36 weeks of age in WT-Veh, POLG-Veh, and POLG-GDF15 mAB2 animals. n = 10/group. Significance was assessed by one-way ANOVA and Tukey HSD. * P < 0.05 .
Absolute body weight of WT-Veh, POLG-Veh, and POLG-GDF15 mAB2 animals at day 1 and day 87 of the study. Treatment starts from day 3. Data are expressed as mean ± standard deviation (SD).

## Slide 2
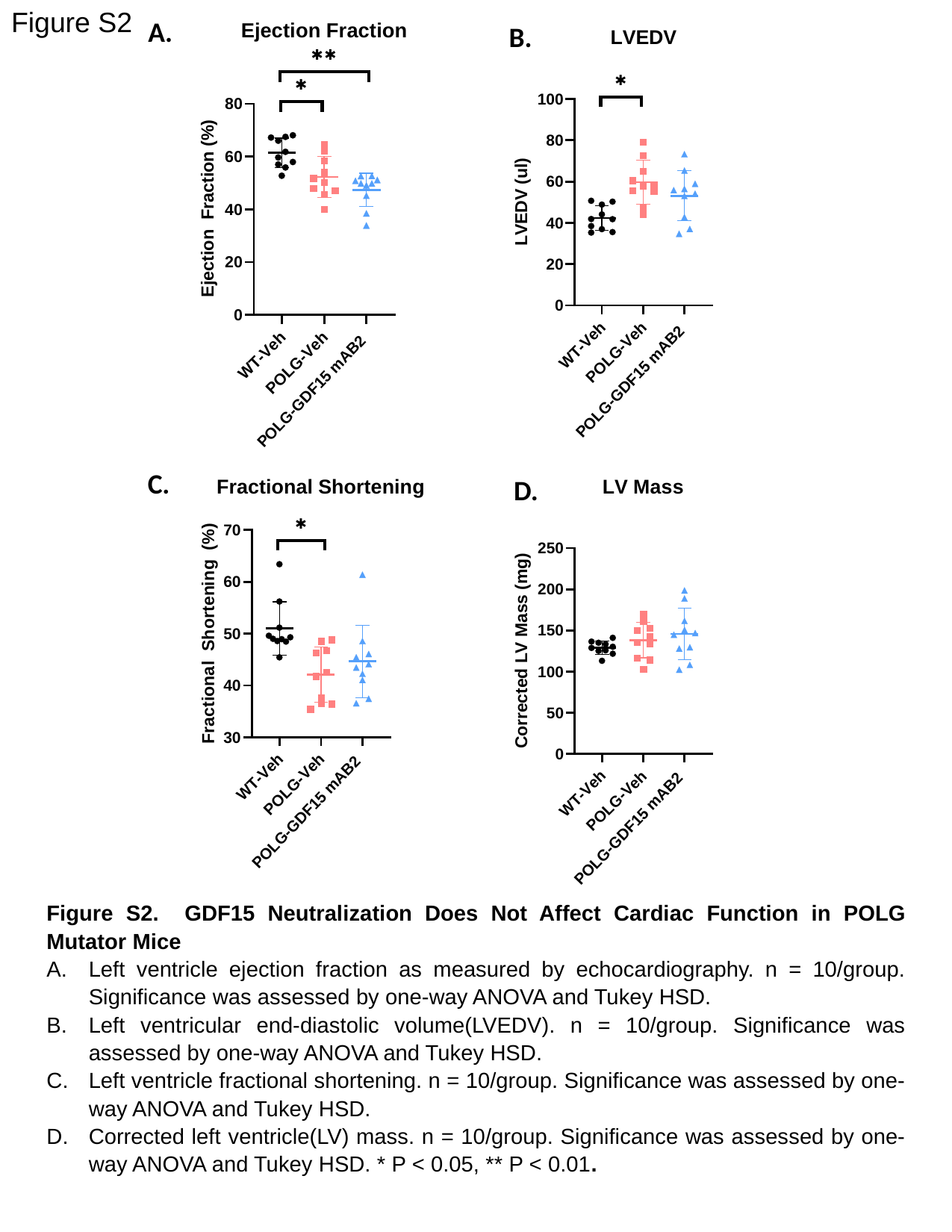

Figure S2
POLG-GDF15 mAB2
A.
POLG-GDF15 mAB2
B.
C.
POLG-GDF15 mAB2
POLG-GDF15 mAB2
D.
Figure S2. GDF15 Neutralization Does Not Affect Cardiac Function in POLG Mutator Mice
Left ventricle ejection fraction as measured by echocardiography. n = 10/group. Significance was assessed by one-way ANOVA and Tukey HSD.
Left ventricular end-diastolic volume(LVEDV). n = 10/group. Significance was assessed by one-way ANOVA and Tukey HSD.
Left ventricle fractional shortening. n = 10/group. Significance was assessed by one-way ANOVA and Tukey HSD.
Corrected left ventricle(LV) mass. n = 10/group. Significance was assessed by one-way ANOVA and Tukey HSD. * P < 0.05, ** P < 0.01.

## Slide 3
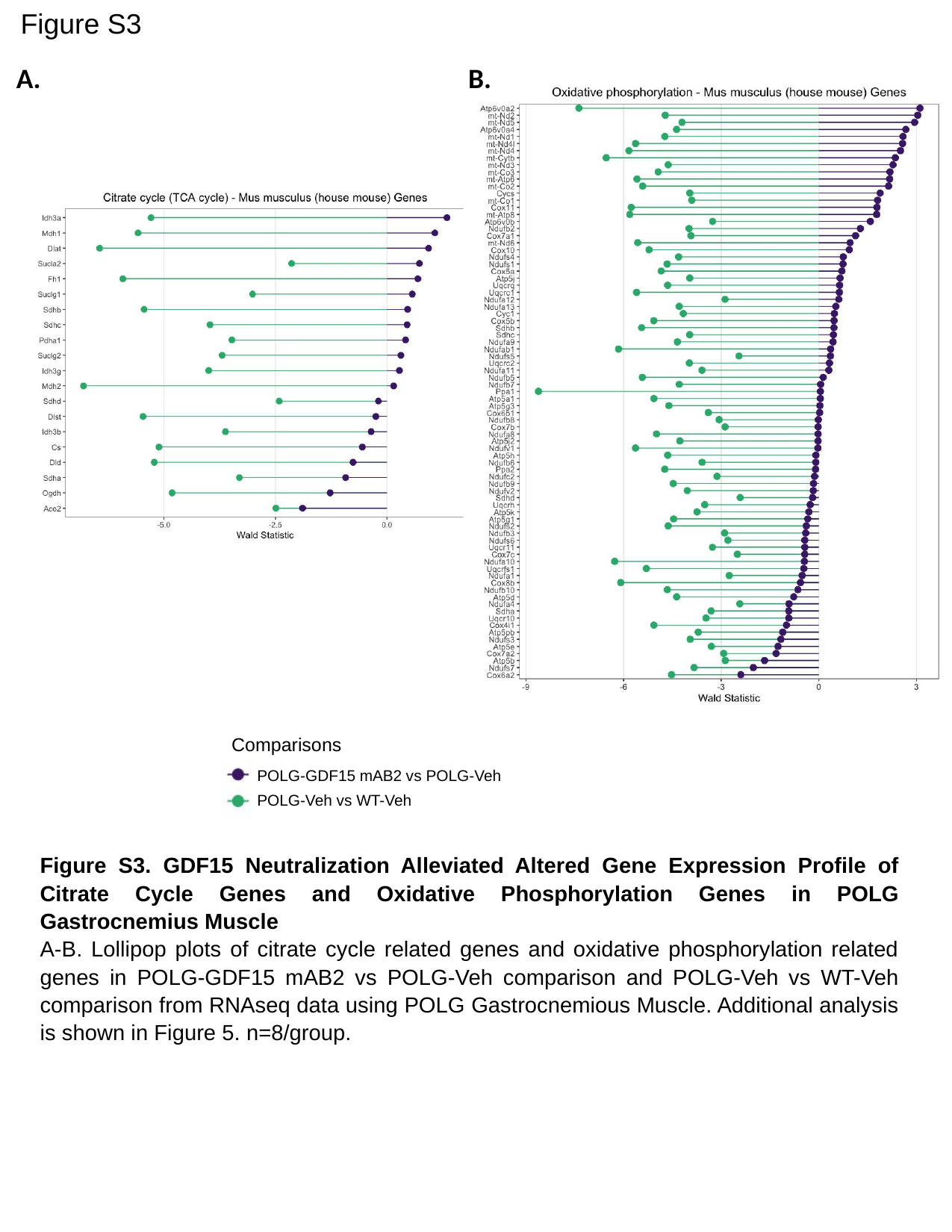

Figure S3
A.
B.
Comparisons
POLG-GDF15 mAB2 vs POLG-Veh
POLG-Veh vs WT-Veh
Figure S3. GDF15 Neutralization Alleviated Altered Gene Expression Profile of Citrate Cycle Genes and Oxidative Phosphorylation Genes in POLG Gastrocnemius Muscle
A-B. Lollipop plots of citrate cycle related genes and oxidative phosphorylation related genes in POLG-GDF15 mAB2 vs POLG-Veh comparison and POLG-Veh vs WT-Veh comparison from RNAseq data using POLG Gastrocnemious Muscle. Additional analysis is shown in Figure 5. n=8/group.
