# Supplementary material for: GDF15 Neutralization Ameliorates Muscle Atrophy and Exercise Intolerance in a Mouse Model of Mitochondrial Myopathy
Source: J Cachexia Sarcopenia Muscle. 2025 Feb 20;16(1):e13715. doi: 10.1002/jcsm.13715 (PMC11840706; doi:10.1002/jcsm.13715)
Supplement: Supplementary file 2 — Data S1 Supplementary References. [file JCSM-16-e13715-s001.docx]

**SUPPLEMENTAL REFERENCES**

S1. Gorman, G.S., et al., *Mitochondrial diseases.* Nat Rev Dis Primers, 2016. **2**: p. 16080.

S2. Murphy, M.P., *How mitochondria produce reactive oxygen species.* Biochem J, 2009. **417**(1): p. 1-13.

S3. Corral-Debrinski, M., et al., *Mitochondrial DNA deletions in human brain: regional variability and increase with advanced age.* Nat Genet, 1992. **2**(4): p. 324-9.

S4. Melov, S., et al., *Multi-organ characterization of mitochondrial genomic rearrangements in ad libitum and caloric restricted mice show striking somatic mitochondrial DNA rearrangements with age.* Nucleic Acids Res, 1997. **25**(5): p. 974-82.

S5. Lee, C.M., et al., *Multiple mitochondrial DNA deletions associated with age in skeletal muscle of rhesus monkeys.* J Gerontol, 1993. **48**(6): p. B201-5.

S6. Wang, Y., et al., *Muscle-specific mutations accumulate with aging in critical human mtDNA control sites for replication.* Proc Natl Acad Sci U S A, 2001. **98**(7): p. 4022-7.

S7. Lax, N.Z., D.M. Turnbull, and A.K. Reeve, *Mitochondrial mutations: newly discovered players in neuronal degeneration.* Neuroscientist, 2011. **17**(6): p. 645-58.

S8. Chen, X., et al., *Mitochondrial dysfunction: roles in skeletal muscle atrophy.* J Transl Med, 2023. **21**(1): p. 503.

S9. Bermejo-Guerrero, L., et al., *Serum GDF-15 Levels Accurately Differentiate Patients with Primary Mitochondrial Myopathy, Manifesting with Exercise Intolerance and Fatigue, from Patients with Chronic Fatigue Syndrome.* J Clin Med, 2023. **12**(6).

S10. Varhaug, K.N., et al., *Serum biomarkers in primary mitochondrial disorders.* Brain Commun, 2021. **3**(1): p. fcaa222.

S11. Kujoth, G.C., et al., *Mitochondrial DNA mutations, oxidative stress, and apoptosis in mammalian aging.* Science, 2005. **309**(5733): p. 481-4.

S12. Wang, D., et al., *GDF15: emerging biology and therapeutic applications for obesity and cardiometabolic disease.* Nat Rev Endocrinol, 2021. **17**(10): p. 592-607.

S13. Emmerson, P.J., et al., *The metabolic effects of GDF15 are mediated by the orphan receptor GFRAL.* Nat Med, 2017. **23**(10): p. 1215-1219.

S14. Johnen, H., et al., *Tumor-induced anorexia and weight loss are mediated by the TGF-beta superfamily cytokine MIC-1.* Nat Med, 2007. **13**(11): p. 1333-40.

S15. Tsai, V.W., et al., *TGF-b superfamily cytokine MIC-1/GDF15 is a physiological appetite and body weight regulator.* PLoS One, 2013. **8**(2): p. e55174.

S16. Yang, L., et al., *GFRAL is the receptor for GDF15 and is required for the anti-obesity effects of the ligand.* Nat Med, 2017. **23**(10): p. 1158-1166.

S17. Breit, S.N., D.A. Brown, and V.W. Tsai, *The GDF15-GFRAL Pathway in Health and Metabolic Disease: Friend or Foe?* Annu Rev Physiol, 2021. **83**: p. 127-151.

S18. Kempf, T., et al., *Prognostic utility of growth differentiation factor-15 in patients with chronic heart failure.* J Am Coll Cardiol, 2007. **50**(11): p. 1054-60.

S19. Husebo, G.R., et al., *Growth differentiation factor-15 is a predictor of important disease outcomes in patients with COPD.* Eur Respir J, 2017. **49**(3).

S20. Lerner, L., et al., *Growth differentiating factor-15 (GDF-15): A potential biomarker and therapeutic target for cancer-associated weight loss.* Oncol Lett, 2016. **12**(5): p. 4219-4223.

S21. Lerner, L., et al., *Plasma growth differentiation factor 15 is associated with weight loss and mortality in cancer patients.* J Cachexia Sarcopenia Muscle, 2015. **6**(4): p. 317-24.

S22. Li, G., et al., *Plasma Growth Differentiation Factor-15 is a Potential Biomarker for Pediatric Pulmonary Arterial Hypertension Associated with Congenital Heart Disease.* Pediatr Cardiol, 2017. **38**(8): p. 1620-1626.

S23. Santos, I., et al., *CXCL5-mediated recruitment of neutrophils into the peritoneal cavity of Gdf15-deficient mice protects against abdominal sepsis.* Proc Natl Acad Sci U S A, 2020. **117**(22): p. 12281-12287.

S24. Hsu, J.Y., et al., *Non-homeostatic body weight regulation through a brainstem-restricted receptor for GDF15.* Nature, 2017. **550**(7675): p. 255-259.

S25. Lerner, L., et al., *MAP3K11/GDF15 axis is a critical driver of cancer cachexia.* J Cachexia Sarcopenia Muscle, 2016. **7**(4): p. 467-82.

S26. Groarke, J.D., et al., *Ponsegromab for the Treatment of Cancer Cachexia.* N Engl J

Med, 2024.

S27. Dobin, A., et al., *STAR: ultrafast universal RNA-seq aligner.* Bioinformatics, 2013. **29**(1): p. 15-21.

S28. Patro, R., et al., *Salmon provides fast and bias-aware quantification of transcript expression.* Nat Methods, 2017. **14**(4): p. 417-419.

S29. Ewels, P., et al., *MultiQC: summarize analysis results for multiple tools and samples in a single report.* Bioinformatics, 2016. **32**(19): p. 3047-8.

S30. Soneson, C., M.I. Love, and M.D. Robinson, *Differential analyses for RNA-seq: transcript-level estimates improve gene-level inferences.* F1000Res, 2015. **4**: p. 1521.

S31. Love, M.I., W. Huber, and S. Anders, *Moderated estimation of fold change and dispersion for RNA-seq data with DESeq2.* Genome Biol, 2014. **15**(12): p. 550.

S32. Wu, T., et al., *clusterProfiler 4.0: A universal enrichment tool for interpreting omics data.* Innovation (Camb), 2021. **2**(3): p. 100141.

S33. Kanehisa, M., *Toward understanding the origin and evolution of cellular organisms.* Protein Sci, 2019. **28**(11): p. 1947-1951.

S34. Kanehisa, M., et al., *KEGG for taxonomy-based analysis of pathways and genomes.* Nucleic Acids Res, 2023. **51**(D1): p. D587-D592.

S35. Kanehisa, M. and S. Goto, *KEGG: kyoto encyclopedia of genes and genomes.* Nucleic Acids Res, 2000. **28**(1): p. 27-30.

S36. Subramanian, A., et al., *Gene set enrichment analysis: a knowledge-based approach for interpreting genome-wide expression profiles.* Proc Natl Acad Sci U S A, 2005. **102**(43): p. 15545-50.

S37. Liberzon, A., et al., *Molecular signatures database (MSigDB) 3.0.* Bioinformatics, 2011. **27**(12): p. 1739-40.

S38. Wall, C.E., et al., *High-fat diet and FGF21 cooperatively promote aerobic thermogenesis in mtDNA mutator mice.* Proc Natl Acad Sci U S A, 2015. **112**(28): p. 8714-9.

S39. Dai, D.F., et al., *Age-dependent cardiomyopathy in mitochondrial mutator mice is attenuated by overexpression of catalase targeted to mitochondria.* Aging Cell, 2010. **9**(4): p. 536-44.

S40. Platt, C., N. Houstis, and A. Rosenzweig, *Using exercise to measure and modify cardiac function.* Cell Metab, 2015. **21**(2): p. 227-236.

S41. Yatsuga, S., et al., *Growth differentiation factor 15 as a useful biomarker for mitochondrial disorders.* Ann Neurol, 2015. **78**(5): p. 814-23.

S42. Piccirillo, R., et al., *Mechanisms of muscle growth and atrophy in mammals and Drosophila.* Dev Dyn, 2014. **243**(2): p. 201-15.

S43. Romanello, V., et al., *Mitochondrial fission and remodelling contributes to muscle atrophy.* EMBO J, 2010. **29**(10): p. 1774-85.

S44. Romanello, V. and M. Sandri, *Implications of mitochondrial fusion and fission in skeletal muscle mass and health.* Semin Cell Dev Biol, 2023. **143**: p. 46-53.

S45. Schakman, O., H. Gilson, and J.P. Thissen, *Mechanisms of glucocorticoid-induced myopathy.* J Endocrinol, 2008. **197**(1): p. 1-10.

S46. Menconi, M., et al., *Role of glucocorticoids in the molecular regulation of muscle wasting.* Crit Care Med, 2007. **35**(9 Suppl): p. S602-8.

S47. Duan, C., et al., *Fully automated mouse echocardiography analysis using deep convolutional neural networks.* Am J Physiol Heart Circ Physiol, 2022. **323**(4): p. H628-H639.

S48. Valero-Breton, M., et al., *Angiotensin-(1-7) improves skeletal muscle regeneration.* Eur J Transl Myol, 2023. **33**(4).

S49. Carter, H.N., et al., *Autophagy and mitophagy flux in young and aged skeletal muscle following chronic contractile activity.* J Physiol, 2018. **596**(16): p. 3567-3584.

S50. Klionsky, D.J., et al., *Guidelines for the use and interpretation of assays for monitoring autophagy (4th edition)(1).* Autophagy, 2021. **17**(1): p. 1-382.

S51. Sanchez, A.M., R. Candau, and H. Bernardi, *Recent Data on Cellular Component Turnover: Focus on Adaptations to Physical Exercise.* Cells, 2019. **8**(6).

S52. Chen, M.L., et al., *Erythroid dysplasia, megaloblastic anemia, and impaired lymphopoiesis arising from mitochondrial dysfunction.* Blood, 2009. **114**(19): p. 4045-53.

S53. Ranjbaran, R., et al., *GDF-15 negatively regulates excess erythropoiesis and its overexpression is involved in erythroid hyperplasia.* Exp Cell Res, 2020. **397**(2): p. 112346.

S54. Penninx, B.W., et al., *Anemia is associated with disability and decreased physical performance and muscle strength in the elderly.* J Am Geriatr Soc, 2004. **52**(5): p. 719-24.
